# The association between later eating rhythm and adiposity in children and adolescents: a systematic review and meta-analysis

**DOI:** 10.1093/nutrit/nuab079

**Published:** 2022-05-04

**Authors:** Mengxuan Zou, Kate Northstone, Rachel Perry, Laura Johnson, Sam Leary

**Affiliations:** NIHR Biomedical Research Centre Nutrition Theme, University of Bristol, Bristol, United Kingdom; Bristol Medical School, Faculty of Health Sciences, University of Bristol, Bristol, United Kingdom; NIHR Biomedical Research Centre Nutrition Theme, University of Bristol, Bristol, United Kingdom; Centre for Exercise, Nutrition and Health Sciences, School of Policy Studies, University of Bristol, Bristol, United Kingdom; NIHR Biomedical Research Centre Nutrition Theme, University of Bristol, Bristol, United Kingdom

**Keywords:** adiposity, adolescence, childhood, later eating rhythm, night eating, obesity, school-age children

## Abstract

**Context:**

Childhood adiposity, an important predictor of adult chronic disease, has been rising dramatically. Later eating rhythm, termed night eating, is increasing in adults but rarely studied in younger ages.

**Objective:**

The objective of this study was to review the association between later eating rhythm and adiposity in children and adolescents. The aspects of later eating being considered included: energy intake (for evening main meal, evening snack, whole evening period, and around bedtime); timing (any food eaten at later timing); and meal frequency in the evening/night (evening main meal skipping, evening snack consumption).

**Data Sources:**

Five databases (the Cochrane Library, CINAHL, Embase, MEDLINE (via OVID), and Web of Science) were searched for eligible articles published prior to and including August 2020.

**Data Extraction:**

Data extraction and quality assessment were conducted by 2 reviewers independently.

**Data Analysis:**

Forty-seven studies were included, all of which were observational. Meta-analysis showed positive associations between both higher energy intake around bedtime (odds ratio [OR] 1.19, 95% CI 1.06, 1.33) and evening main meal skipping (OR 1.30, 95% CI 1.14, 1.48), and adiposity. There was evidence to suggest that consuming evening snacks reduced adiposity, but it was very weak (OR 0.80, 95% CI 0.62, 1.05). No association was seen between eating later and adiposity (OR 1.04, 95% CI 0.68, 1.61). In the narrative analysis, approximately half of the studies suggested that there was no association between later eating rhythm and adiposity, either as a whole or within exposure subsets.

**Conclusion:**

The magnitude of the relationship between later eating rhythm and adiposity is very small, and may vary depending on which aspects of later eating rhythm are under consideration; however, the evidence for this conclusion is of very low certainty . Further research with a more consistent definition of “later timing”, and longitudinal studies in different populations, may lead to different conclusions.

**Systematic Review Registration:**

PROSPERO registration no. CRD42019134187.

## INTRODUCTION

Rates of childhood adiposity have increased dramatically in the last few decades,^1^^,^^2^ and it has been shown to be an important predictor of adulthood chronic diseases.^3–7^ Furthermore, childhood adiposity is associated with enormous financial burden for national health-care systems.^8^ Thus, childhood adiposity has been considered as one of the most serious public health issues in the 21st century by the World Health Organization (WHO).^9^

Eating habits are identified as one of the key modifiable lifestyle behaviors for preventing childhood adiposity.^10^ As the continuity and stability of eating behavior traits tend to run throughout childhood and adulthood,^11^ developing healthy eating habits early in life could be an efficient method for reducing the likelihood of developing diet-related diseases in adulthood.^12–14^ Recent studies have linked circadian rhythm, as well as timing-related factors, to adiposity, by demonstrating changes in energy regulation through circadian-driven processes, such as transport of lipids, glucose, and dietary proteins in the intestine.^15–20^ Time-related factors such as duration of sleep and breakfast skipping have been reported to have an impact on childhood adiposity.^21–24^ These reports emphasize the potentially important role of time at which food is consumed during the day in relation to adiposity development. Notably, energy intake (EI) during the nighttime, relative to that during other time periods, has been highlighted as of particular concern.

“Night eating” (NE) is a term that has been used in previous studies to describe food intake occurring during the evening and night in children.^25^ This term was first proposed by Stunkard et al^26^ more than 50 years ago, but no consistent definition has yet been agreed upon. The majority of studies describe NE as encompassing 3 aspects: (1) timing of food consumption, (2) amount of EI after a given time and (3) meal frequency (ie, frequency of evening meal or snacks),^26–33^ eg, consuming over 25% of total daily energy intake (TDEI) after 7 pm on all recording days. However, instead of using the more restrictive term NE, there are a substantial number of studies describing eating patterns in the evening/night covering one aspect only of NE such as timing, EI, or meal frequency. These studies have used terms such as “later eating,” “nocturnal eating,” “late-night overeating” and “nighttime EI.” To harmonize the inconsistent use of terms and to cover later eating behaviors comprehensively from all perspectives, a broader term “later eating rhythm” encompassing NE and eating more in the later part of the day has been adopted here.The possible mechanism relating later eating to increased weight has been investigated at the physiological level. Studies have shown that adults exhibit less-efficient energy metabolism and decreased whole-body fat oxidation during the evening.^34–36^ On the other hand, emerging studies in animals have shown that a wide variety of metabolic markers (such as adipokines, glucocorticoids, and clock genes) are affected by later eating, thereby increasing fat storage and weight gain.^37^^,^^38^ In addition, patterns of EI distribution across the day varied by countries and global regions ; however, food and beverage intake in the evening tends to be more energy dense, and dinner is estimated to be the largest main meal (compared with breakfast and lunch) in most high-income countries.^39–41^ Moreover, late-night overeating has been shown to be associated with breakfast skipping and shorter sleep duration in children.^32^^,^^42^^,^^71^ As noted above, time-related factors such as duration of sleep and breakfast skipping, have been confirmed to have an impact on childhood adiposity, although it is unclear whether eating later per se is the causal factor, or whether other correlated factors are driving this association.^37^ Therefore, later eating rhythm is likely to be important for preventing childhood adiposity but, to our knowledge, the evidence in children has never been systematically reviewed.

A recent systematic review investigating the association between a larger dinner and excess weight in adults included 10 observational studies and 8 clinical trials. The meta-analysis of 4 observational studies showed weak evidence of a positive association between evening eating and body mass index (BMI), with a mean difference of −0.39 kg/m^2^ (95% CI −0.80 kg/m^2^ to 0.01 kg/m^2^); however, no evidence of association was shown in the meta-analysis of 5 clinical trials (mean difference −0.89 kg/m^2^, 95% CI −2.52 kg/m^2^ to 0.75 kg/m^2^).^39^ The only review including all age groups found that the EI distribution over the day varied by country and geographical area.^41^ In the narrative analysis of 10 observational studies (4 studies in children/adolescents and 6 in adults), the authors speculated that higher evening EI may be a major risk factor for obesity in all populations, because the majority of studies showed a positive association. However, this review also reported that it was difficult to draw definitive conclusions due to the high heterogeneity of the populations, sample sizes, and assessment methods for diet and weight status between studies. Overall, the results of the existing reviews appear to challenge the commonly held belief “breakfast like a king, lunch like a prince, and dinner like a pauper” which recommends a reduction in energy intake across the day for weight management.^44^ However, the evidence for this is insufficient in children/adolescents. There are currently no recommendations for the optimal distribution of EI across the day for children. Given the recognized physiological effect of NE on adiposity and the limited evidence from population-based studies, a comprehensive systematic review of the relationship between later eating rhythm and adiposity in children and adolescents is needed to fill gaps in the knowledge.

The objective of this review was to evaluate the association between later eating rhythm and adiposity in children and adolescents, in terms of timing of food consumption, EI after 4 pm, and frequency of meal consumption in the evening/night.

## METHODS

### Selection criteria

This review has been reported in accordance with the Systematic Reviews and Meta-Analyses (PRISMA) guidelines.^45^ The review protocol has been published^46^ and is also accessible through PROSPERO (registration no. CRD42019134187).^47^ The eligibility criteria for included papers are listed as follows, according to PICOS (Table 1):

**Table 1:** PICOS criteria for inclusion of studies

Parameter	Criterion
Population	Children and adolescents
Interventions/exposures	Night eating
Comparisons	Non–night eating
Outcomes	Adiposity (body mass index, waist circumference, fat mass index, and waist-to-hip ratio)
Study design	Randomized controlled trials and observational studies

#### Study design.

Randomized controlled trials (RCTs) and observational studies (cohort studies, cross-sectional studies, and case–control studies) were included in the search strategy. Studies had to be original research. Reviews, case studies, and surveys were excluded, but the references of any review papers were searched for further studies.

#### Participants.

Studies involving children or adolescents aged 4–18 years old were included. Studies with participants who were critically ill, or who had endocrine disorders or syndromic obesity, were excluded.

#### Intervention/exposure.

NE was the intervention/exposure of interest. As noted in the Introduction, there are different ways to define NE; however, they consider 3 aspects: timing, EI, and meal frequency. This review considered all studies in which the intervention/exposure was later meal or snack time in the evening or at nighttime (defined as 4 pm–11.59 pm); diet in which a greater proportion of TDEI or absolute higher EI was consumed in the evening/night; relatively more meal/snack/drink occasions occurred in the evening.

In terms of dietary assessment, this review included studies using 24-hour food recall with at least 1 recorded day, food diary with at least 1 recorded day, direct observation, and/or food frequency questionnaires (FFQs).

#### Comparison.

In accordance with the intervention/exposure, the comparison was non-NE which was classified as: earlier meal or snack time in the evening/night (4 pm–11.59 pm); diet in which a smaller proportion of TDEI was consumed in the evening/night; fewer meal/snack/drink occasions in the evening.

#### Outcomes.

Studies have been included if they reported at least 1 of the following measurements of childhood adiposity: BMI/BMI standard deviation score (BMI-SDS) or BMI *z*-score; waist circumference (WC); fat mass index (FMI)/percentage of body fat (%BF); waist-to-hip ratio (WHR).

### Search strategy

A systematic search of both published and unpublished literature was conducted up until August 2020 with the assistance of an experienced systematic reviewer (R.P.) using 5 electronic databases: the Cochrane Library, CINAHL, Embase, MEDLINE (via OVID), and Web of Science. An example of the search strategy for use in MEDLINE is shown in the Supporting Information (see Appendix S1 in the Supporting Information online). The search strategy for each database was similar but revised appropriately to take into account any differences in controlled vocabulary and syntax rules. The reference lists of all included articles as well as relevant review articles were hand-searched for further studies. Conference papers and abstracts were used to help identify potential articles, and authors were contacted to see whether full-text articles were available. Studies in all languages were included. Non-English articles were translated where possible. Searches were carried out 3 times, including the initial search on November 2018, updated searching on November 2019, and searching prior to submission.

### Study records

The EndNote reference management software package was used to manage all the records. All duplicates were removed. The titles and abstracts were initially screened by one reviewer (M.Z.), and double screening was carried out by the other members of the review team. Studies that did not meet the inclusion criteria were excluded.

The full texts of potentially relevant articles were retrieved (M.Z. and R.P.) and reviewed independently by the authors. Reasons for exclusion were recorded and reported in Table S1 in the Supporting Information online. One reviewer (M.Z.) extracted data from all included papers, with double extraction carried out by the other reviewers (K.N., R.P., and S.L.). The extraction form was designed specifically for this study and was piloted by all reviewers based on the first 3 papers identified. Any disagreement between reviewers was resolved through discussion with the third reviewer (K.N.).

### Quality assessment

Each included article was assessed for methodological quality and risk of bias by 2 of the reviewers independently; again, discrepancies were resolved through discussion with the third reviewer. The Newcastle–Ottawa scale,^48^ a star system (with a maximum of 9 stars), was used to assess the risk of bias of cohort studies and case–control studies, in which the quality of studies was assessed from 3 aspects: selection of the study groups, comparability of the study groups, and ascertainment of either the exposure or outcome of interest. Owing to limited quality assessments being available for cross-sectional studies, the adapted Newcastle–Ottawa scale (with a maximum of 10 stars) was used.^49^ The adaptions included: (1) enquiry about the general representativeness of the whole sample instead of the exposure group and control groups separately, and (2) the criteria “Demonstration that the outcome of interest was not present at start of study” was removed because this was not relevant (Table S2 in the Supporting Information online).

### Statistical analysis

The associations between later eating rhythm and adiposity were analyzed through meta-analyses, and the results of other studies were discussed through narrative synthesis. Given the variety of definitions of NE (7 exposures), studies included in the current review were divided into 3 categories: *timing of food consumption* (eating at later timing), *energy intake* (EI for evening main meal; EI for evening snack; EI for whole evening; EI around bedtime), and *meal frequency* (evening main meal skipping; evening snack consumption).

Studies included in the meta-analysis were required to

1. report data at a level of detail sufficient for the pooled analysis,
2. have a plausibly similar definition/measurement of exposures and outcome, and
3. reach the minimum number (3) of studies in each subset.

The detailed reasons for exclusion from meta-analysis are presented in Table S3 in the Supporting Information online. As a result, 4 primary meta-analyses were conducted on the association between 4 exposures and overweight/obesity:

1. eating at later timing (the definition of later timing varied in different studies but “later than 10 pm” and “within 2 hours before bedtime” were mainly used),
2. higher EI around bedtime,
3. skipping the evening main meal, and
4. consuming evening snacks.

Studies were included in the meta-analysis if the outcome was overweight/obesity categorized from BMI, based on any of the following definitions: the International Obesity Task Force (IOTF), Cole et al,^50^ the World Health Organization (WHO), or the 85th percentile of national growth charts or its corresponding cut-off points. Odds ratios (ORs) and confidence intervals (CIs) were extracted from the included studies when available. Where these were not reported, the unadjusted ORs were calculated by hand from the reported event numbers in exposure and control groups. Adjusted ORs were selected over unadjusted ORs, and where multiple adjusted ORs were reported in the same study, the OR for the most-adjusted model was chosen.^51^

Stata (version 15.0) was used to conduct the meta-analyses. Random-effects models rather than fixed-effects models were used where heterogeneity was considerable (*I*^2^ statistic greater than 50%).^51^ The likelihood of publication bias was tested through visual inspection of funnel plots and by performing Egger’s regression test. The strength of the overall body of evidence for each meta-analysis was assessed using the Grading of Recommendations Assessment, Development and Evaluation (GRADE) methodology.^52^ Subgroup analyses in separate age or sex categories were unlikely to be conducted due to insufficient data. Similarly, subgroup analyses on different study types were not able to be performed.

Owing to the high heterogeneity in terms of the type and measurements of later eating rhythm and adiposity, the harvest plot method was applied to visually display the results of all studies in the narrative synthesis. This method is novel and is useful for synthesizing the best available evidence across a heterogenous group of studies.^53^ For each exposure, 4 bar charts were created to indicate the associations of each exposure with each outcome (overweight; obesity, and overweight/obesity, each of which was categorized by (a) BMI, and (b) adiposity measured by any other eligible measurement). Each bar chart consists of 3 columns representing the direction of the association, and each bar represents 1 study. The following 3 characteristics are also presented in the harvest plot: (1) the quality of each study, indicated by the height of the bar; (2) studies conducted among children are indicated with gray bars, and those conducted among adolescents with black bars; and (3) studies included in the meta-analysis are annotated with a star on the top of the respective bars.

### Sensitivity analysis

To examine the robustness of the meta-analysis findings, sensitivity analyses excluding low-quality studies were conducted. The main analyses using adjusted ORs and unadjusted ORs were repeated, separately, due to the inconsistency in the confounders that were adjusted for across the different studies. The main analyses using alternative-effects models were also performed.

## RESULTS

### Study characteristics

**Figure 1:**
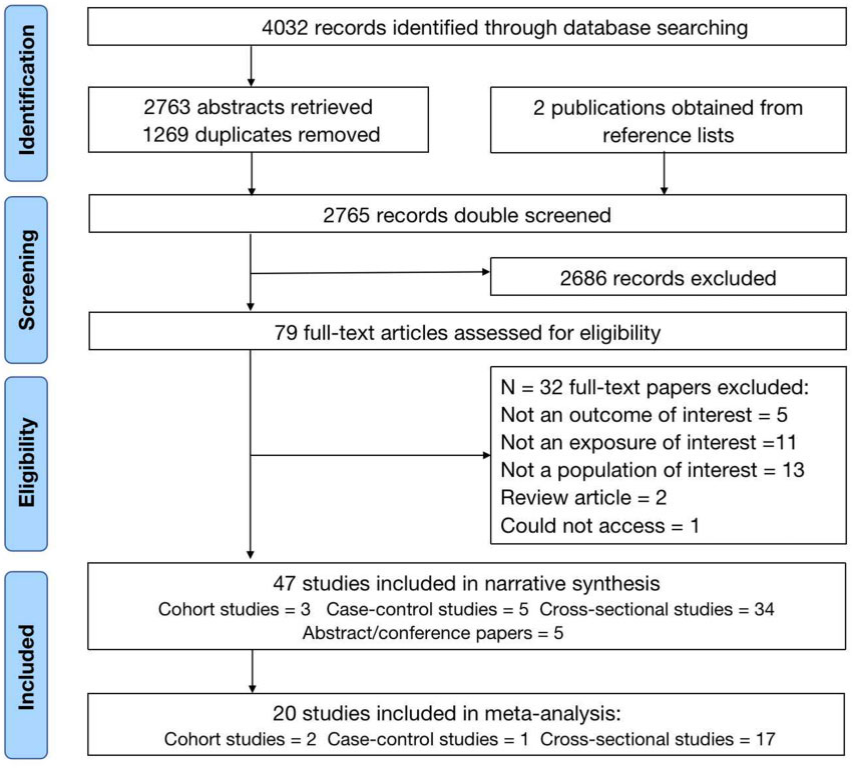
Preferred Reporting Items for Systematic Reviews and Meta-Analyses (PRISMA) flow diagram.

Of the 2765 studies identified, a total of 47^25^^,^^33^^,^^43^^,^^54–97^ studies were included, consisting of 42 full-text studies of 73,450 children/adolescents in 30 countries, plus 5 abstract/conference papers^55^^,^^61^^,^^73^^,^^81^^,^^92^ of 35,790 children/adolescents in 5 countries. The numbers of studies that were excluded at each stage are shown in Figure 1.

For the 47 studies included in the review, the number of participants varied from 8 to 24,885, ages ranged from 2 to 18 years old, and 51.7% were girls. Sixteen studies focused on children (2–10 years), 25 on adolescents (11–18 years), and 6 studies on both. Most included both sexes, with only 3 studies^58^^,^^87^^,^^96^ reporting gender-specific ORs. Four studies focused on girls only,^59^^,^^74^^,^^90^^,^^91^ and 1 on boys only^75^; 3 abstract papers did not clarify gender.^55^^,^^73^^,^^92^ Study populations were from Europe (*n* = 26), Australia (*n* = 1), North America (*n* = 7), South America (*n* = 3), the Middle East (*n* = 3), Asia (*n* = 10), and Africa (*n* = 2). The studies were published between 1980 and 2020. Of the 42 full-text studies, 34 were cross-sectional studies, 5 were case–control studies, and 3 were cohort studies. No RCTs were found. Detailed characteristics of each full-text study can be found in Table 2.^25^^,^^33^^,^^43^^,^^54^^,^^56–60^^,^^62–72^^,^^74–80^^,^^82–91^^,^^93–97^

**Table 2:** Characteristics of 42 included full-text studies, ordered by author

Study	Study design	Country of study Year of study/follow-up (years)	Study sample			Dietary assessment method	Criteria of later eating rhythm				Exposure	Outcome	Result				Adjustment variables
			Number in analysis	Age (years) Mean (SD)/Range	%Female		Later timing	Higher energy intake	Evening meal skipping	Evening snack consumption			Unadjusted OR (95% CI) overweight/obese vs normal	Adjusted OR (95% CI) overweight/obese vs normal	Other effect size/*P* value for BMI	Effect size/*P* value for outcomes other than BMI	
Agustina et al^a^ (2020)^91^	CS	Indonesia 2016	324	12–19	100.0	24-hour dietary recall for 2 d	^b^	^b^	Yes	^b^	Dinner skipping (23.1%): skipping meals between 1700 h and 0000 h	BMI: overweight/obesity (BMI > 1 SD) defined by WHO (17%)	Weekday: 1.88 (1.01–3.52)^f^ Weekend: 1.23 (0.68–2.39)^f^	Weekday: 2.06 (1.07–3.99)^f^ Weekend: 1.22 (0.63–2.35)^f^	^b^	^b^	Energy intake, age, mother’s education level, frequency of listening to radio
Alavi et al (2006)^54^	CC	Thailand^b^	70 (36 cases, 34 controls)	10–12	38.8	Self-reported questionnaire	Yes	^b^	^b^	^b^	Eating snack before going to bed investigated on weekdays and weekend days separately	BMI *z*-score: obesity (*z*-score above 3 SD)	Weekday: 0.70 (0.27 to 1.80)^d^ Weekend: 1.11 (0.43 to 2.85)^d^	^b^	^b^	^b^	^b^
Azadbakht et al^a^ (2019)^85^	CS	Iran 2009–2010	5642	10–18	49.9	Self-reported questionnaire	^b^	^b^	Yes	^b^	Dinner skipping (25%): Consuming dinner <5 times per week	BMI: BMI; overweight/obesity (BMI ≥ 85th percentile) defined by WHO WC: abdominal obesity (waist-to-height ratio > 0.5)	1.62 (1.39 to 1.89)^f^	1.53 (1.22 to 1.94)^f^	BMI (kg/m^2^): Dinner consumer: 19.30 ± 3.97 Dinner skipper: 19.79 ± 4.42 *P* < .001	Abdominal obesity: Unadjusted: 1.59 (1.36–1.85)^f^ Adjusted: 1.65 (1.30–2.10)^f^	Age, sex, family socio-economic position (parental occupation, parental education, private car ownership, school type [public/private], home type [private/rented] and home personal computer), PA, and smoking
Azizi et al (2001)^78^	CS	Iran^b^	421	10–19	58.0	24-hour dietary recall for 2 usual days	^b^	Yes	^b^	^b^	% of TEI for dinner and evening snack separately^h^	BMI: overweight/obesity (BMI ≥ 85th percentile) defined by Must et al, 1991	^b^	^b^	T-test: Energy intake for dinner: Boys: *P* > 0.05; girls: *P* > .05 Energy intake for evening snack: Boys: *P* > .05; girls: (2.5% vs 1.7%) *P* < .05	^b^	^b^
Barbu et al (2015)^33^	CS	Romania 2011	886	6–18	53.2	Self-reported questionnaire	Yes	^b^	^b^	^b^	Last meal later than 2200 h: 94.5% in younger children and 85.2% in adolescents	BMI: overweight/obesity defined by WHO criteria	^b^	^b^	^b^	^b^	^b^
Ben Slama et al (2002)^93^	CC	Tunisia^b^	167 (95 cases, 72 controls)	6–10	49.0	Self-reported questionnaire completed by parents	^b^	^b^	^b^	Yes	Eating evening snack	BMI: Obesity (BMI ≥ 97th percentile) by M.F. Rolland–Cachera reference curves (3.9% in boys; 3.4% in girls)	7.97 (4.19 to 15.15)^d^	^b^	^b^	^b^	^b^
Bo et al^a^ (2014)^66^	CS	Italy 2011	400	11–13	48.0	Self-reported questionnaire by parents	^b^	Yes	^b^	^b^	Highest energy intake from snacks derived from evening snack (44.75%)	BMI: overweight/obesity (BMI ≥ 85th percentile)	2.31 (0.97 to 5.51)^d^^,^^f^	3.12 (1.17 to 8.34)^e^^,^^f^	^b^	^b^	Age, sex, PA, total calories intake
Bodur et al^a^ (2010)^56^	CS	Turkey^b^	496	12–15	45.0	Self-reported questionnaire	Yes	^b^	^b^	^b^	Eating before going to bed: From time to time 354 (71.37%) Daily 142 (28.63%)	BMI: overweight/obesity (BMI ≥ 85th percentile) (21.6%)	1.68 (1.07 to 2.64)^d^^,^^f^	2.70 (1.30 to 5.70)^f^	^b^	^b^	PA, preference of cookies, chips; not consuming dried fruit daily, eating dessert regularly, having breakfast irregularly
Choi et al^a^ (2017)^57^	CS	Korea 2012	688	15 (0.8)	50.9	Self-reported questionnaire	Yes	^b^	^b^	^b^	Frequency of eating food 2 h after dinner: More than 6 times/week 30 (4.4); 4–5 times/week 62 (9); 2–3 times/week 155 (22.5); Once/week or less 167 (24.3); Almost not 274 (39.8)	BMI: overweight (23–24.9); obesity (BMI > 25) by WHO definitions	0.59 (0.34 to 1.03)^d^^,^^f^	^b^	^b^	^b^	^b^
Ciccone et al^a^ (2013)^95^	CS	Canada 2006	1008	6–8	48.3	Self-reported questionnaire	^b^	^b^	^b^	Yes	Eating evening snack (62%) Male: 59.3% Female: 65.2%	BMI: overweight; obesity defined by WHO criteria	0.76 (0.58 to 0.99)^d^^,^^f^	^b^	^b^	^b^	^b^
Coulthard and Pot^a^ (2016)^58^	CS	UK 2008–2012	1620 in total Children (gp 1) *n* = 768 Adolescents (gp 2) *n* = 852	Gp1: 4–10 Gp2: 11–18	Gp1: 48.4 Gp2: 49.9	Self-reported food diaries for 4 consecutive days	Yes	^b^	^b^	^b^	Timing of evening meal (g1: G 2) 2000–2159 (3.3%:3.3%) 1700–1959 (83.3%:83.3%) 1400–1659 (13.4%:14.3%)	BMI: overweight/obesity (85th percentile cut-off) with UK90 charts	^b^	Children (g1): Boys: 1.64 (0.37 to 7.26) Girls: 1.11 (0.34 to 3.59) Total: 1.33 (0.53 to 3.33)^f^ Adolescents (g2): Boys: 1.02 (0.48 to 2.18) Girls: 0.71 (0.35 to 1.42) Total: 0.83 (0.50 to 1.38)^f^	^b^	^b^	Sex, household income, ethnicity
De Cnop et al^a^ (2018)^79^	CS	Brazil 2010	1749	10–19	50.1	Self-reported questionnaire	^b^	^b^	Yes	^b^	Dinner skipping (Consuming dinner < 7 times per week)	BMI: overweight/obesity (BMI > 1 SD), used distribution curves by WHO WC: overweight/obesity (WHR > 0.5) % of body mass: overweight/obesity (>25% for boys and >30% for girls)	^b^	Public school 1.03 (0.74 to 1.73)^f^ Private school 1.22 (0.93 to 1.60)^f^	^b^	% fat mass criteria: Public school 1.24 (0.87 to 1.75)^f^ Private school 1.35 (1.03 to 1.78)^f^ WHR criteria: Public school: 1.12 (0.6 to 1.82)^f^ Private school: 1.52 (1.05 to 2.22)^f^	Sex and age
Dmitruk et al^a^ (2018)^59^	CS	Poland 2015	151	16–18	100.0	Self-reported questionnaire	Yes	^b^	^b^	^b^	Last meal timing later than 2000 h	BMI: overweight/obesity defined by Cole et al. WHR: abdominal obesity (WHR > 0.8)	4.58 (2.18 to 9.62)^d^^,^^f^	^b^	^b^	WHR: χ^2^ = 17.84, *P* < .001	^b^
Dubois et al (2009)^76^	CS	Canada 2002	1520	44 mos–56 mos	49.0	Interviewed 24-hour dietary recall	^b^	Yes	^b^	^b^	Energy intake (kcal) for dinner (1700–1900)^h^	BMI: mean	^b^	^b^	^b^ (No data, linear graph suggested positive relationship in breakfast skipper; not found in breakfast eaters)	^b^	^b^
Abd El-Shaheed et al^a^ (2019)^60^	CC	Egypt^b^	90 (45 cases, 45 controls)	10–18	65.6	Interviewed questionnaire	Yes	^b^	^b^	^b^	Sleep after dinner by less than 2 h: No (64%), sometimes (10.1%), yes (25.8%)	BMI: overweight/obesity (BMI ≥ 85th percentile)	Sleep after dinner by less than 2 h: 0.78 (0.34 to 1.78)^d^^,^^f^	^b^	^b^	^b^	^b^
Eloranta et al (2012)^77^	CS	Finland 2007–2009	510	6–8	48.0	4-d food diary for 4 consecutive days (including 1 or 2 weekend days)	^b^	Yes	^b^	^b^	% of TEI for dinner^h^	BMI: overweight/obesity defined by IOTF criteria; WC (cm); Hip circumference (cm); % body mass	^b^	0.96 (0.92 to 1.00)^e^^,^^f^	^b^	WC (cm): β = −0.01 *P* = .914 Hip circumference (cm): β = −0.01 *P* = .860 % body mass: β = 0.01 *P* = .781	Age, sex, PA, screen time, and parental income.
Eng et al (2009)^43^	CS	NHANES in US 1999–2004	11072	Gp 1: 2–5 Gp 2: 6–11 Gp 3: 12–18	49.0	24-hour dietary recall for 1 d	^b^	Yes	^b^	^b^	% of TEI in 2-h intervals from 1600 h to midnight^h^	BMI: Overweight (95th > BMI ≥ 85th percentile); obesity (BMI ≥ 95th percentile)	^b^	^b^	Overweight: Total: β = 0.20, *P* = .967 Children: β = 16.70, *P* = .007 Adolescents: β = −15.90, *P* = .009 obesity: Total: β = 3.00, *P* = .552 Children: β = 10.30, *P* = .184 Adolescents: β = −5.20, *P* = .518	^b^	Age, gender, ethnicity, and Linear Time Trend: a 4-level discreet time trend variable was created to indicate dietary energy intake at: Time point 0: 1600 to 1759 time point 1: 1800 to 1959 time point 2: 2000 to 2159 time point 3: 2200 to 2359
Fayet et al (2012)^70^	CS	Australia 2007	4837	Gp 1: 2–5 Gp 2: 6–11 Gp 3: 12–18	^b^	24-hour dietary recall for 2 nonconsecutive days	^b^	Yes	^b^	^b^	% of TEI for dinner (1700–2030) (32% in children, 30% in adolescents) and evening snack (2030+) (4.6% in children and 11% in adolescents), separately^h^	BMI: overweight; obesity defined by CDC in US	^b^	^b^	^b^(No significant relationship in children, but *P* value was not reported.)	^b^	^b^
Gómez-Martínez et al (2012)^88^	CS	Spain 2000–2002	1978	13–18.5	51.4	Self-reported questionnaire	^b^	^b^	Yes	Yes	Dinner skipping (irregular dinner) Evening snack consumption (usually)	Sum of 6 skinfolds (mm)^h^ WC (cm)^h^	^b^	^b^	^b^	Dinner skipping *P* > .2 for all models Evening snack *P* > .05 for all models	Model 1: age Model 2: age and PA
Hernandez et al^a^ (2016)^67^	CS	Korea 2010–2012	1738	12–18	45.4	24-hour dietary recall for 1 d	Yes	Yes	^b^	^b^	NE (consuming ≥25% TEI during 2100 h–0600 h for 1 recorded day) NE (20.8%) Non-NE (79.2%)	BMI: BMI *z*-score; overweight (95th > BMI ≥ 85th percentile); obesity (BMI ≥ 95th percentile)	1.03 (0.78 to 1.35)^d^^,^^f^	Overweight: 0.90 (0.54 to 1.40) Obesity: 1.13 (0.65 to 1.98)	Total: β = 0.18, *P* = .007 Boys: β = 0.11, *P* < .001 Girls: β = 0.28, *P* = .004	^b^	Logistic regression model: age, sex, total calories intake Liner regression model: plus PA, <8 h average sleep per night; high stress levels
Karatzi et al (2017)^71^	CS	Greece 2007	1912	9–13	50.1	24-hour recall for 2 consecutive weekdays and 1 weekend day	^b^	Yes	^b^	^b^	Energy intake (kcal) for dinner (482 kcal), and dinner and evening snack (545.9 kcal), separately^h^	BMI: BMI *z*-score	^b^	^b^	Dinner: Total: β = 0.03, *P* = .214 low PA: β = 0.11, *P* = .019 High PA: β = −0.083, *P* = .081 Dinner and evening snack: Total: β = 0.004, *P* = .878 Low PA: β = 0.07, *P* = .105 High PA: β = −0.1, *P* = .033	^b^	Age, gender, and tanner stage
Lamerz et al^a^ (2005)^25^	CS	Germany 2002–2003	1979	5–7	49.1	Self-reported questionnaire by parents	Yes	Yes	^b^	^b^	NE – getting up and eating high calorie food in the evening or night at least 1 time/week for a time period of at least 3 mos: NE (1.1%) Non-NE (98.9%)	BMI: overweight/obesity (BMI ≥ 90th percentile) (9%)	1.02 (0.23 to 4.38)^d^^,^^f^	^b^	^b^	^b^	^b^
Lehto et al (2011)^80^	CS	Finland 2006	604	9.6	51.7	Self-reported questionnaire	^b^	^b^	Yes	^b^	Dinner skipping (Consuming dinner <5 times per school week)	BMI: 17.5 (17.3 to 17.7) kg/m^2^	^b^	^b^	Model 1: β = −0.38 (−0.98 to 0.23) Model 2: β = −0.45 (−1.05 to 0.15) Model 3: β = −0.45 (−1.1 to 0.15)	^b^	Model 1: age + sex Model 2: plus sleep duration on school week, PA and screen time, dietary pattern scores Model 3: plus parental employment and family structure
Lioret et al (2008)^86^	CS	France 1998–1999	721 in total Younger children (gp 1) *n* = 331 Older children (gp 2) *n* = 390	Gp 1: 3–6 Gp 2: 7–11	^b^	7-d food diary	^b^	^b^	Yes	^b^	Dinner skipping (Consuming dinner <7 times per week): 10.7% in total; gp1 (10.3%); gp2 (11%)	BMI: overweight defined by IOTF criteria.	3–11 yrs 0.89 (0.46 to 1.70)^d^	^b^	^b^	^b^	^b^
Maffeis et al (2000)^72^	CS	Italy^b^	530	7–11	47.5	Interviewed questionnaire	^b^	Yes	^b^	^b^	% of TEI for dinner (28.3% for boys, 27.2% for girls) and evening snack (2.4% for boys and 1.8% for girls), separately^h^	% fat mass	^b^	^b^	^b^	Dinner and % fat mass: Boys *r* = 0.15 *P* < .05 Girls *r* = 0.11 *P* > .05 Both sex *r* = 0.10 *P* < .05 Evening snack and % fat mass: Boys *r *= −0.19 *P* < .01 Girls *r* = −0.11 *P* > .05 Both sex *r* = −0.17 *P* < .001	^b^
Musaiger et al^a^ (2014)^87^	CS	Bahrain 2006–2007	735	15–18	53.9	Self-reported questionnaire	^b^	^b^	Yes	Yes	Dinner skipping Mid-night snack consumption (sometimes/always)	BMI: overweight/obesity (BMI ≥ 85th percentile)	Dinner skipping Boys: 0.86 (0.47 to 1.61)^f^ Girls: 0.80 (0.51 to 1.27)^f^ Total: 1.12 (0.79 to 1.57)^f^^,^^g^ Mid-night snack consumption 0.98 (0.71 to 1.34)^f^^,^^g^	^b^	^b^	^b^	^b^
Ochiai et al^a^ (2013)^96^	CS	Japan 1999–2008	3128	12–13	49.3	Self-reported questionnaire	^b^	^b^	^b^	Yes	Snacking after dinner: (seldom/none; always/often)	BMI: overweight/obesity defined by IOTF criteria. (14.56 % in boys, 11.6% in girls)	0.98 (0.79 to 1.21)^d^^,^^f^	^b^	Boys Seldom or none 42.6% Always or often 57.4% *P* = .441 Girls Seldom or none 41.5% Always or often 58.5% *P* = .700	^b^	^b^
Ostachowska-Gasior (2016)^84^	CS	Poland 2013–2014	3009	13–17	55.1	Self-reported questionnaire	^b^	^b^	Yes	^b^	BMI (kg/m^2^)	Dinner skipping (Consuming dinner <3 times per week) (11.5% for girls, 4.3% for boys)	^b^	1.03 (0.99 to 1.09)^c^	^b^	^b^	Age, sex, breakfast skipping, second breakfast skipping (small meal before midday), dessert skipping
Reed et al (2013)^90^	CS	US 2010	43	10–12	100.0	Self-reported questionnaire	^b^	^b^	Yes	^b^	Number of dinners consumed in a week	BMI: Overweight (95th > BMI ≥ 85th percentile); obesity (BMI ≥ 95th percentile)	^b^	^b^	Variance test: Normal weight: 6.88 (0.42) Overweight 6.17 (1.3) Obesity 6.12 (1.85) *P* > .05	^b^	^b^
Rychkova et al (2019)^62^	CC	Buryatia 2016	158 (79 cases, 79 controls)	11–17	35.4	Self-reported questionnaire	Yes	^b^	^b^	Yes	Eating before going to bed; Evening meal/snack consumption	BMI: obesity (BMI ≥ 95th percentile)	Eating before going to bed: 1.66 (0.89 to 3.12)^d^ Eating at night: 1.16 (0.40 to 3.37)^d^	^b^	^b^	^b^	^b^
Sun et al^a^ (2020)^97^	CS	China 2012	2085	10.83 (0.993)	46.3	Self-reported questionnaire	^b^	^b^	^b^	Yes	Late-night snacks consumption (55.1%)	BMI: overweight/obesity (BMI > 1 SD) defined by WHO (25.3%)	0.98 (0.79 to 1.21)^d^^,^^f^	^b^	^b^	^b^	^b^
Thompson et al (2006)^74^	Cohort study	US Baseline–1990 2–10 follow-up years (median = 6)	Baseline–196 End–101	Baseline: 8–12 End: 11–19	100.0	7-d food diary for 7 consecutive days at baseline and follow-up	^b^	Yes	Yes	^b^	% of TDEI from 1700 h to 0559 h at baseline^h^: weekdays: 39% (16.5–57.2) weekend: 41.0% (0.0–68.3) Combined: 40.2% (16–59.3) Main meal frequency from 1700 h to 0559 h at baseline (<2 times; 2–3 times; >3 times) Weekdays: 1.4 (0.75–3.8) weekend: 1.5 (0.0–5.0) combined: 1.6 (0.9–3.6)	BMI: the change of BMI *z*-score between baseline and follow-up	^b^	^b^	Energy intake Weekdays: β = 1.41 *P* = .039 Weekends: β = −0.01 *P* = NS Combined: β = 1.15 *P* = NS Meal frequency weekdays: <2 times vs >3 times *P* = .0471 weekend: *P* = NS combined: *P* = NS	^b^	Baseline BMI *z*-score
Band and Tepe^a^ (2019)^94^	CS	Turkey 2019	791	11–13	51.3	Self-reported questionnaire	^b^	^b^	^b^	Yes	Eating evening snack (sometimes/always)	BMI: overweight/obesity (BMI > 1 SD) defined by WHO (46.4%)	0.51 (0.38 to 0.68)^d^^,^^f^	^b^	^b^	^b^	^b^
Vik et al (2013)^89^	CS	Belgium, Greece, Hungary, the Netherlands, Norway, Slovenia and Spain 2010	7915	11.5	52.0	Self-reported questionnaire	^b^	^b^	Yes	^b^	Dinner skipping yesterday (7%): Belgium 1%, Greece 18%, Hungary 8%, the Netherlands 2%, Norway 3%, Slovenia 12%, and Spain 3%	BMI: overweight (18%); obesity (5%) defined by IOTF criteria	^b^	^b^	χ^2^ test: prevalence of dinner skipping in each group: Normal weight (5%) Overweight (11%) Obesity (21%) *P* < .001	^b^	^b^
Vilela et al^a^ (2019)^68^	Cohort study	Portugal 2005–2012 4 years follow-up	1961	4 at baseline	48.9	3-d food diary (2 weekdays and 1 weekend day)	^b^	Yes	^b^	^b^	Lunch and evening pattern: relatively higher energy intake at lunch and supper, which implies late eating pattern Energy intake (kcal) for dinner (1900–2130) and supper (2130-), separately^h^	BMI: overweight/obesity defined by WHO criteria	1.14 (1.04–1.25)^f^	1.21 (1.06–1.37)^f^	*t*-test for the relationship between energy intake in various meals and weight status. Dinner: *P* = .435 Supper: *P* = .281	^b^	Parental education, TEI, maternal age and education, children’s *z*-score BMI, number of eating occasions, Children’s Eating Behavior Questionnaire subquestions
Watanabe et al (2016)^63^	CS	Japan 2003	1545	3–6	46.6	Self-reported questionnaire	Yes	^b^	^b^	^b^	Relatively late dinner timing: 6 clusters referred to different pattern of behavior regarding timing of dinner, sleeping duration, and screen time	BMI: overweight defined by IOTF criteria	^b^	^b^	*P* value comparing 6 clusters: Unadjusted: *P* = .007 Adjusted: *P* = .010	^b^	Family socio-economic position, family environments, parents’ behaviors, such as meal regularity, habitual exercise, screen time
Waxman and Stunkard (1980)^75^	CC	United States^b^	8 (4 cases, 4 controls)	Obese group: 7, 11, 13, 4.5 Non-obese group: 6, 10, 12, 5.5	0.0	Direct observation by observers: the size and number of portions	^b^	Yes	^b^	^b^	Energy intake (kcal) for dinner^h^ Obese group: 766 (290) Non-obese group: 504 (1183)	Weight: obesity (97th percentile for weight on Wetzel Grid)	^b^	^b^	F[1, 33] = 23.42, *P* < .001	^b^	^b^
Wijtzes et al^a^ (2016)^82^	Cohort study	Netherland^b^ follow up from 4 years to 6 years	5913	Baseline: 4 years End of follow-up: 6 years	50.3	Self-reported questionnaire at both baseline and end	^b^	^b^	Yes	^b^	Dinner skipping (Consuming dinner <7 times per week): At 4 yrs 7.1% in total, 7.9% in boys, 6.2% in girls At 6 yrs 3.1% in total, 3.5% in boys, 2.8% in girls From 4 yrs to 6 yrs Stable dinner skipping 21 (0.6%)	BMI: overweight/obesity defined by IOTF criteria at 6 yrs; % fat mass at 6 yrs;	Dinner skipping at 4 yrs 1.22 (0.71 to 2.09)^f^ Dinner skipping at 6 yrs 1.63 (1.14–2.34)^f^ Stable dinner skipping 1.10 (0.32–3.75)^f^	(Models 1–3 and full model) Dinner skipping at 4 yrs^f^ (0.57 to 1.74) 0.83 (0.47 to 1.47) 0.84 (0.49 to 1.43) 0.87 (0.48 to 1.60) Dinner skipping at 6 yrs^f^ 1.36 (0.90 to 2.05) 1.10 (0.69 to 1.76) 1.12 (0.70 to 1.79) Stable dinner skipping^f^ 1.17 (0.31–3.35) 0.94 (0.24–3.61) 0.95 (0.24–3.68) 0.34 (0.05–2.20)		Fat mass (crude model, model 1–3, and full model); β (95% CI) Dinner skipping at 4 yrs 0.55 (−0.66 to 1.76) 0.37 (−0.58 to 1.31) 0.09 (−1.10 to 0.91) 0.14 (−1.11 to 0.83) 0.04 (−0.69 to 0.77) Dinner skipping at 6 yrs 1.83 (1.00 to 2.65) 1.51 (0.73 to 2.29) 0.86 (−0.08 to 1.79) 0.89 (−0.04 to 1.81)	Model 1: sex, age, family socio-economic position, ethnic background, and parental BMI. Model 2: model 1 + other meal skipping behaviors at age 4 yrs. Model 3: model 2 + children’s lifestyle behaviors. Full model: model 3 + BMI at age 4 yrs.
Yoo et al^a^ (2015)^64^	CS	Korea 2006	2004	9.42 (1.65)	53.2	Self-reported questionnaire	Yes	^b^	^b^	^b^	Eating before going to bed (22.9%)	BMI: underweight (BMI < 5th percentile); overweight/obesity (BMI ≥ 85th percentile)	0.68 (0.52 to 0.89)^d^^,^^f^	0.28 (0.01 to 0.93)	^b^	^b^	Age, gender
Yorulmaz and Pacal (2012)^83^	CS	Turkey^b^	250	16.9 (0.87)	49.6	Self-reported questionnaire	^b^	^b^	Yes	^b^	Dinner skipping (9.2%)	BMI percentile *n* (%): <5th percentile 4 (1.6) 5–15th percentile 39 (15.6) 15–85th percentile 170 (68) 85–95th percentile 34 (13.6) ≥96th percentile 3 (1.2)	^b^	^b^	χ^2^ test *P* > .05	^b^	^b^
Yüksel et al (2017)^69^	CS	Turkey 2017	859	15.9 (1.3)	18.6	Interviewed NE questionnaire	Yes	Yes	^b^	^b^	NE syndrome (high calories intake at late night) score (0–52) NE (scored >25; 21.1%)	BMI: overweight (85–95 percentile) (19.2%), obesity (>95th percentile) (13.7%)	^b^	^b^	Association between NE score and BMI: *P* > .050 (No other data were reported.)	^b^	^b^
Zalewska and Maciorkowska^a^ (2017)^65^	CS	Poland 2011	1832	18	65.2	Self-reported questionnaire	Yes	^b^	Yes	^b^	Time of supper: <2000 h; ≥2000 h (later timing) Dinner skipping	BMI: overweight/obesity (BMI ≥ 85th percentile) defined by WHO criteria.	Timing: 0.58 (0.43 to 0.77)^d^^,^^f^ Dinner skipping: 1.35 (0.90 to 2.03)^d^^,^^f^	^b^	^b^	^b^	^b^

a Studies included in meta-analysis.

b Information not available/not calculable.

c Transformed odds ratio (OR) used in meta-analysis.

d Unadjusted odds ratio (OR)/confidence interval (CI) calculated from reported event number.

e Risk ratio.

f Overweight and obese.

g Calculation combining gender.

h Continuous variable.

Abbreviations: BMI, body mass index; CC, case control study; CDC, Centre for Disease and Control; CS, cross-sectional study; gp, group; IOTF, International Obesity Task Force; NE, night eating; PA, physical activity; TDEI, total daily energy intake; TEI, total energy intake; UK90, UK growth reference chart; WC, waist circumference; WHO, world health organization; WHR, waist-to-hip ratio.

All the studies fitted into 7 exposures developed from the 3 aspects of later eating rhythm. Five of the studies examined 2 exposures each.^62^^,^^65^^,^^74^^,^^87^^,^^88^ The numbers and study designs of studies included in the narrative synthesis and the meta-analysis by exposures are shown in Figure S1 in the Supporting Information online. Adiposity was commonly measured by overweight/obesity, followed by overweight and obesity, which were categorized by BMI. Very few studies used BMI/BMI *z*-score or other measurements (FMI, WC). Twenty studies were included in the meta-analyses examining the relationship between 4 of the exposures and overweight/obesity. The reasons why studies were excluded from the meta-analyses are listed in Table S3 in the Supporting Information online.

### Quality assessment

Table 3 ^25^ ^,^ ^33^ ^,^ ^55^ ^,^ ^57–61^ ^,^ ^63–74^ ^,^ ^76–82^ ^,^ ^84–93^ ^,^ ^95–99^ outlines the quality assessment of the full-text studies included in the review. Seven studies^66–68^^,^^77^^,^^80^^,^^85^^,^^91^ were described as high quality, losing no more than 2 stars across the 3 domains: sample selection, comparability, and outcome. Twenty-seven studies were described as medium quality, and 8 studies^57^^,^^59^^,^^60^^,^^69^^,^^83^^,^^84^^,^^90^^,^^93^ were described as low quality.

**Table 3:** Quality assessment of 42 included full-text studies

Study:	Selection (max 5 stars)	Comparability (max 2 stars)	Outcome (max 3 stars)	Total/max^a^	Quality^b^
Authors (year)					
Cross-sectional studies					
Agustina et al (2020)^91^	5	1	3	8/10	High
Azadbakht et al (2019)^85^	4	2	3	9/10	High
Azizi F et al (2001)^78^	3	0	3	6/10	Medium
Barbu et al (2015)^33^	4	0	2	6/10	Medium
Bo et al (2014)^66^	5	2	3	10/10	High
Bodur et al (2010)^56^	3	1	3	7/10	Medium
Choi et al (2017)^57^	3	0	0	3/10	Low
Ciccone et al (2013)^95^	3	1	3	7/10	Medium
Coulthard and Pot (2016)^58^	3	1	3	7/10	Medium
De Cnop et al (2018)^79^	3	1	3	7/10	Medium
Dmitruk et al (2018)^59^	0	0	2	2/10	Low
Dubois et al (2009)^76^	4	0	2	6/10	Medium
Eloranta et al (2012)^77^	3	2	3	8/10	High
Eng et al (2009)^43^	3	1	3	7/10	Medium
Fayet et al (2012)^70^	3	0	2	5/10	Medium
Gómez-Martínez et al (2012)^88^	2	1	3	6/10	Medium
Hernandez et al (2016)^67^	3	2	3	8/10	High
Karatzi et al (2017)^71^	3	2	2	7/10	Medium
Lamerz et al (2005)^25^	4	0	2	6/10	Medium
Lehto et al (2011)^80^	3	2	3	8/10	High
Lioret et al (2008)^86^	4	0	1	5/10	Medium
Maffeis et al (2000)^72^	3	1	3	7/10	Medium
Musaiger et al (2014)^87^	3	0	3	6/10	Medium
Ochiai et al (2013)^96^	2	0	3	5/10	Medium
Ostachowska-Gasior (2016)^84^	1	1	2	4/10	Low
Reed et al (2012)^90^	2	0	0	2/10	Low
Sun et al (2020)^97^	3	0	3	6/10	Medium
Band and Tepe et al (2019)^94^	3	0	3	6/10	Medium
Vik et al (2013)^89^	3	0	3	7/10	Medium
Watanabe et al (2016)^63^	2	1	3	6/10	Medium
Yoo et al (2015)^64^	4	1	2	7/10	Medium
Yorulmaz and Pacal (2012)^83^	1	0	0	1/10	Low
Yüksel et al (2017)^69^	1	0	2	3/10	Low
Zalewska and Maciorkowska (2017)^65^	3	0	3	6/10	Medium
Case–control studies					
Alavi et al (2006)^54^	2	0	2	4/9	Medium
Ben Slama et al (2002)^93^	2	0	1	3/9	Low
Abd El-Shaheed et al (2019)^60^	1	0	2	3/9	Low
Rychkova et al (2017)^62^	2	1	2	5/9	Medium
Waxman and Stunkard (1980)^75^	1	1	2	4/9	Medium
Cohort studies					
Thompson et al (2006)^74^	2	0	2	4/9	Medium
Vilela et al (2019)^68^	3	1	3	7/9	High
Wijtzes et al (2016)^82^	2	1	2	5/9	Medium

a Quality assessment forms were designed specifically for each type of study design, with a maximum of 10 stars for cross-sectional studies and 9 stars for cohort/case–control studies.

b The quality of studies are rated as: High (8–10), Medium (5–7), or Low (0–4) for cross-sectional studies, and as High (7–9), Medium (4–6), or Low (0–3) for cohort/case–control studies.

Most studies (35/42) selected their samples from a community setting that was representative of the general population. Dietary measurement varied according to the type of exposure variable. Studies assessing timing of food consumption tended to use self-reported (9/11) or interview-administered (1/11) questionnaires; only 1 study used a recorded 4-day food diary.^58^ For EI, the majority of studies (10/14) used methods with higher reliability and validity, such as food diaries recorded for multiple days (eg, for 3 days,^68^ 4 days,^77^ or 7 days^74^), 24-hour food recall for 1 day^43^^,^^67^^,^^76^ or 2 days,^70^^,^^71^^,^^78^ or direct observation.^75^ Almost all studies assessing meal frequency used self-reported (parent or child) questionnaires (19/22), and only 2 studies used food diaries recorded for 7 days.^74^^,^^86^ The majority of studies (38/42) assessed adiposity using independent measurements (measured weight and/or height or waist/hip circumference); the remaining studies used self-reported weight and/or height. Of the 34 studies reporting overweight/obesity based on BMI, half of them (17/34) used commonly approved international criteria such as IOTF criteria (6/34), WHO criteria (10/34), or cut-offs defined by Cole et al^50^ (1/34). However, almost all the others (15/32) used cut-off points in accordance with the 85th/95th percentile or greater than one third standard deviation of national growth charts. The 2 remaining studies used 90th percentile and 97th percentile to define overweight and obesity, respectively.^25^^,^^93^ Nearly half of the studies (18/42) adjusted for confounders. Age (13/18), sex (12/18), socio-economic status (9/18), ethnicity (7/18), and physical activity (7/18) were the most common confounders used for adjustment. Other confounders such as total EI (TEI) (5/18), baseline BMI (5/18), other meal regularity (4/18), sleeping quality (2/18), and parental eating behavior (2/18) were less common. Eight studies presented both adjusted and unadjusted results; however, most of them (6/8) did not show substantial differences between adjusted and unadjusted results.

### Meta-analysis and descriptive analysis

#### Timing of food consumption and adiposity

**Figure 2:**
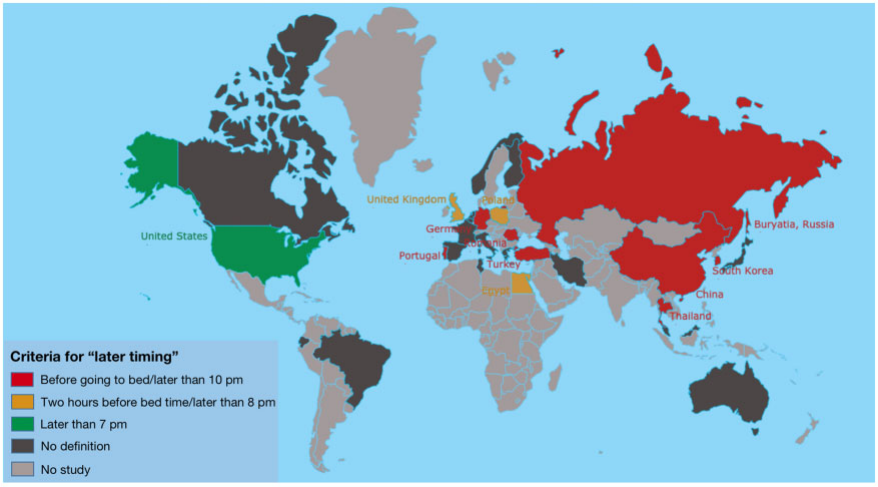
The criteria for eating at “later timing” in children/adolescents for the included studies.

The association between timing of food intake and adiposity was investigated in 13 studies (Figure S1 in the Supporting Information online).^33^^,^^54–65^ The definitions of later timing varied among studies, with 5^54^^,^^56^^,^^61^^,^^62^^,^^64^ defining it as “eating before sleeping,” 3^58^^,^^59^^,^^65^ as “last meal later than 8 pm,” and the rest as “dinner within 2 h before sleep,”^60^ “eating 2 hours after dinner,”^57^ “eating after 7 pm,”^55^ or “last meal later than 10 pm.”^33^ The prevalence of eating later in the evening was lowest (3.3%) in the United Kingdom,^58^ with other countries ranging from 22.9%^64^ to 95.5%^33^ (Table 2). The definitions of later timing by countries are shown in Figure 2, indicating that Western countries tended to use an earlier time for “later timing” criteria compared with Eastern countries.

**Figure 3:**
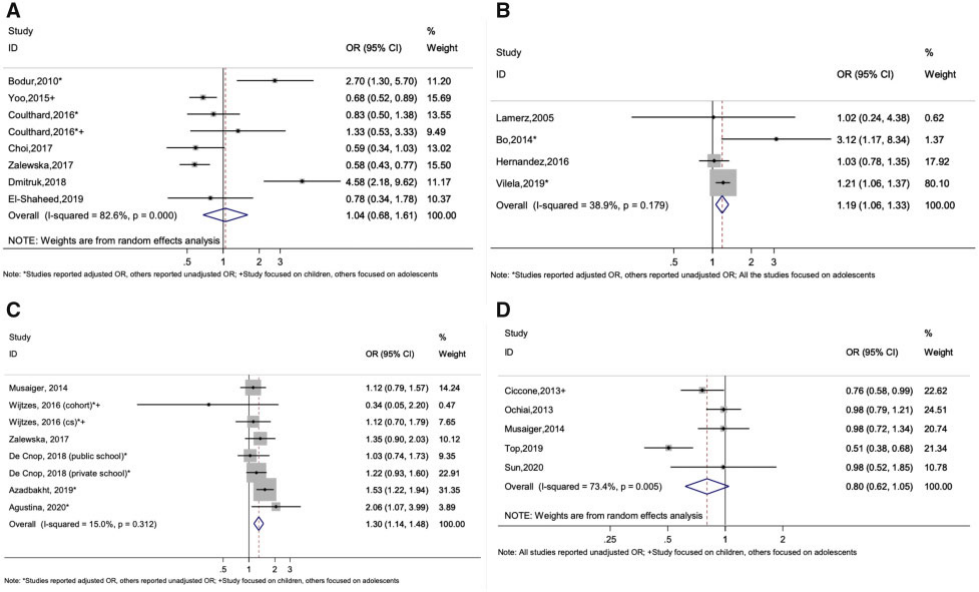
Meta-analyses of (A) the relationship between eating at later timing (after 8 pm in general) and adiposity, using unadjusted and adjusted ORs (7 unique studies); (B) the relationship between higher energy intake at later timing and adiposity, using unadjusted and adjusted ORs (4 unique studies); (C) the relationship between evening meal skipping and adiposity, using unadjusted and adjusted ORs (6 unique studies); and (D) the relationship between evening snack consumption and adiposity, using unadjusted ORs (5 unique studies).

The meta-analysis was based on the 7 studies^56–60^^,^^64^^,^^65^ that compared the odds of overweight/obesity in those who ate later in the evening compared with those who ate earlier, and yielded a pooled OR of 1.04 (95% CI 0.68 to 1.61). There was substantial statistical heterogeneity, with an *I*^2^ of 82.6% (*P* < .001) (Figure  3A^57–61^^,^^65^^,^^66^). According to the GRADE system, the certainty of the evidence was very low (see Table S4 in the Supporting Information online).

**Figure 4:**
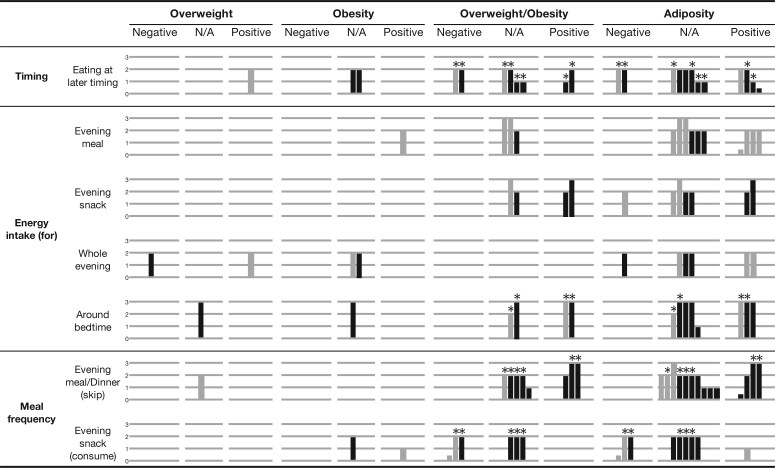
**Summary of the evidence from all the included studies (*n* = 47) for the associations between later eating rhythm and adiposity.** A “supermatrix” covering all categories of exposures, consisting of 7 rows (each row representing a dimension of later eating rhythm related to 3 aspects: timing, energy intake, and meal frequency) and 3 columns for each weight category (the 3 columns representing the 3 possible directions of the associations between each of the indicators of later eating rhythm and weight status: negative association, no association (N/A), or positive association). Each bar represents an association between later eating rhythm and weight status. Studies conducted among populations of children (≤10 years) are indicated with half-tone (gray) bars, and studies conducted among populations of adolescents (>10 years) are indicated with full-tone (black) bars. The quality of each study is indicated by the height of the bar (3 = high quality; 2 = medium quality; 1 = low quality; and 0.5 = abstract/conference paper). The studies included in the meta-analysis are indicated by a star on the top of each bar.

Six studies could not be included in the meta-analysis because they reported on either overweight^63^ or obesity,^54^^,^^62^ rather than overweight/obesity, or they lacked sufficient data (Table S3 in the Supporting Information online).^33^^,^^55^^,^^61^ Overall, as shown in the harvest plot in Figure 4, half of the studies with relatively high quality did not find strong associations between eating at later timing and adiposity.

No gender differences were reported by the only study^58^ that stratified the analysis by gender. No clear differences were found between children and adolescents (Figure 4). No meaningful differences were found between adjusted and unadjusted results due to the small subset of available studies, with only 4/13 adjusting for confounders. In addition, 2 studies^56^^,^^64^ reported results before and after adjustment, but neither of them reported substantial differences (see Table S5 in the Supporting Information online).

#### Energy intake and adiposity.

The association between EI in the evening/night and adiposity was reported in 15 studies.^25^^,^^43^^,^^66–78^ The majority of studies (10/15) used continuous exposures, which fitted into 3 exposure categories: EI for evening main meal (*n* = 9),^68^^,^^70–73^^,^^75–78^ EI for evening snack (*n* = 5),^66^^,^^68^^,^^70^^,^^72^^,^^78^ and EI for the whole evening (after 4 pm) (*n* = 3).^43^^,^^71^^,^^74^ EI was only presented in 3 studies focusing on the evening main meal, with 2 studies using percentage of TEI (27.75%^72^ and 31%^70^ of TEI) and 1^71^ study using absolute energy (482 kcal). The remaining 5 studies^25^^,^^66–69^ (5/15) used the categorical exposure NE, which referred to higher EI around bedtime: “having most energy-dense snack for evening snack”,^66^ “consuming over 25% of TEI after 9 pm”,^67^ “consuming higher energy after 9.30 pm”,^68^ “high calories intake at late night”,^69^ or “getting up and eating high-calorie food at night.”^25^ The prevalence of NE decreased from 44.75%^66^ to 1.1%^25^ when restricting the criteria of NE to later timing and higher EI (Figure S1 in the Supporting Information online).

The meta-analysis of 4 studies^25^^,^^66–68^ reporting the odds of overweight/obesity in adolescents who had higher EI around bedtime (described as night eaters in studies) vs non–night eaters is shown in Figure  3B.^25^^,^^66–68^ There was low statistical heterogeneity (*I*^2^ = 38.9%, *P* = .179). The pooled OR of 1.19 (95% CI 1.06 to 1.33) demonstrated an elevated odds of overweight/obesity among adolescents who were night eaters (consuming higher EI at later timing) compared with those who were not. According to the GRADE system, the certainty of the evidence was very low (see Table S4 in the Supporting Information online).

Eleven studies could not be included in any meta-analyses due to insufficient data^69^ or inconsistent exposures (Table S3 in the Supporting Information online).^43^^,^^70–78^ Overall, as shown in Figure 4, approximately half of the studies did not find strong associations between any of the exposures of EI and adiposity.

Contrary results were seen with respect to associations for children and adolescents: positive associations between EI for main evening meal, EI for the whole evening, and adiposity were suggested by most studies in children. Positive association between EI for evening snack and adiposity was only seen in adolescents. A total of 7 studies (7/15) adjusted for confounders when focusing on all exposure groups of EI; the proportion of positive associations was slightly higher in the adjusted results (6/13) compared with in the unadjusted results (6/21). Four studies^66–68^^,^^77^ reported results before and after adjustment, with only one^65^ reporting a difference. However, the subsets of studies focusing on EI were too few to show meaningful differences between adjusted and unadjusted results, with only 1 to 3 studies adjusting for confounders within each exposure group (see Table S5 in the Supporting Information online).

#### Meal frequency and adiposity.

The association between meal frequency and adiposity was reported in 22 studies^62^^,^^65^^,^^74^^,^^79–97^: 12 used evening meal skipping as the exposure, 7 used evening snack consumption, 2^87^^,^^88^ used both exposures, and only 1 study^74^ used the main meal frequency after 5 pm.

#### Evening meal skipping.

Fourteen studies reported on evening meal skipping (Figure S1 in the Supporting Information online).^65^^,^^79–91^ The prevalence of skipping evening meals ranged from 3.1%^82^ to 25%^85^ for the majority of studies, but there was a much higher prevalence of 63.3% in 1 study.^81^

Six studies^65^^,^^79^^,^^82^^,^^85^^,^^87^^,^^91^ were included in the meta-analysis. There was very low statistical heterogeneity (*I*^2^ = 15.0%, *P = .*312). The pooled OR for childhood adiposity in relation to evening meal skipping was 1.30 (95% CI 1.14 to 1.48), demonstrating an elevated odds of overweight/obese children who skipped evening meals compared with those who consumed evening meals regularly (Figure  3C^65^^,^^79^^,^^82^^,^^85^^,^^87^^,^^91^). According to the GRADE system, the certainty of the evidence was very low (see Table S4 in the Supporting Information online).

Eight studies were excluded from the meta-analysis due to not reporting overweight/obesity^84^^,^^86^ or to having insufficient data (Table S3 in the Supporting Information online).^80^^,^^81^^,^^83^^,^^84^^,^^86^^,^^88–90^ Overall, as shown in Figure 4, no evidence of associations between evening main meal skipping and adiposity were found in most studies (10/14).

No gender differences were found in the only study that undertook subgroup analysis by gender. Slight differences were found between children and adolescents, with positive associations suggested only in adolescents. No substantial differences were observed between adjusted and unadjusted results, with slightly more than half of the adjusted results (8/12) suggesting no association, and the outcome of the analysis was similar for the unadjusted results (6/12). Three studies^82^^,^^85^^,^^91^ reported results before and after adjustment, with only 1^82^ reporting an observed difference (see Table S5 in the Supporting Information online).

#### Evening snack consumption.

Nine studies focused on evening snack consumption (Figure S1 in the Supporting Information online).^62^^,^^87^^,^^88^^,^^92–97^ The prevalence of evening snack consumption was reported in only 2 studies^95^^,^^97^ with 55.1% and 62.0%, respectively.

The meta-analysis was based on 5 studies,^87^^,^^94–97^ and yielded a pooled OR of 0.80 (95% CI 0.62 to 1.05), suggesting that there may be a reduction in adiposity if evening snacks are consumed. There was substantial statistical heterogeneity, with an *I*^2^ of 73.4% (*P* = .005) (Figure  3D^87^^,^^94–97^). According to the GRADE system, the certainty of the evidence was very low (see Table S4 in the Supporting Information online).

The remaining studies were excluded from the meta-analysis because they reported on obesity only^62^^,^^93^ or WC^88^ rather than overweight/obesity, or because they did not present sufficient data (Table S3 in the Supporting Information online).^92^ Overall, as shown in Figure 4, the majority of studies (5/9) did not find a strong association between evening snack consumption and adiposity.

However, contrary results were seen with respect to associations for children and adolescents, with most studies in children suggesting negative associations. No gender differences were found in studies^87^^,^^96^ that reported results for males and females separately. No meaningful differences were found between adjusted and unadjusted results due to the small subset of studies, with only 1^88^ adjusting for confounders (see Table S5 in the Supporting Information online).

Only 1 cohort study^74^ investigated main meal frequency after 5 pm and reported no association with adiposity.

### Sensitivity analysis

The meta-analysis was repeated for later eating after excluding the low-quality studies.^57^^,^^59^^,^^60^ This reduced the heterogeneity across studies, but the findings for association between eating later and adiposity were similar (see Figure S2 in supporting information online). The quality of studies focusing on other exposures was moderately high, so there was no need for any further sensitivity analysis. All the main analyses were repeated using adjusted ORs and unadjusted ORs separately where applicable, and the findings were similar (Figures S3–S5 in the Supporting Information online). The main analyses were repeated using alternative-effects models, and the findings were similar except for a negative association being suggested between later eating and adiposity (see Figures S6–S9 in the Supporting Information online).

### Small study effects

Visual assessment of the funnel plots suggested no strong evidence of publication bias (Egger *P = .*062; 0.679; 0.308; 0.873) for any of the main meta-analyses (see Figure S10 in the Supporting Information online).

## DISCUSSION

### Main findings

To our knowledge, this is the first systematic review examining the relationship between later eating rhythm and adiposity in children and adolescents. The results of this review suggested that children/adolescents who consumed relatively higher energy at later timing (around bedtime) or those who skipped the evening main meal were more likely to be overweight/obese compared with those who did not. These findings may seem contradictory . However, none of the studies focusing on evening main meal skipping adjusted for TEI. It may therefore imply that the consequence of skipping the evening main meal may be higher EI later on, around bedtime. As a result, this could increase TDEI, thereby increasing the odds of adiposity. The relationship between consumption of evening snacks and adiposity was uncertain, as no consistent associations were found (very weak statistical evidence of an association was found in the meta-analyses, but negative associations were reported in most studies of children, and no strong associations were reported in most studies of adolescents). No statistical evidence of associations between timing (eating at a later timing) and adiposity were found, although positive associations were observed in most studies that focused on children only. It was difficult to draw a definitive conclusion regarding the association between EI in the evening and adiposity, as the various studies concentrated on different eating occasions (evening main meal; evening snack; whole evening period; around bedtime) and had conflicting results; however, age differences were observed, positive associations were more likely to be seen in children than adolescents within most subsets of EI. Overall, no substantial differences were seen between the adjusted and unadjusted results; however, the level of association remains uncertain within each exposure group due to the small subsets of studies. The authors have very little confidence in these results due to the very low certainty of the evidence according to GRADE.

### Comparison with other studies

The findings of this review can be compared with those of a previous review in adults by Fong et al,^39^ and 2 previous reviews in both adults and children, 1 by Almoosawi et al^41^ and the other by Lopez-Minguez et al.^98^ Similarly to 2 of the previous published reviews, the considerable inconsistency, not only in the definition of NE, but also in the aspects that could contribute an effect, such as meal timing and the EI, that were considered. For example, when defining “later timing” of food intake, the current review found that Western countries tended to set an earlier time for the criterion of later timing of food intake compared with Eastern countries (see Figure 2), which implied that children in Western countries tended to have earlier eating/sleeping habits compared with Eastern countries. Similarly, Lopez-Minguez et al^98^ also found that the time criteria differed from country to country. For example, in Europe, Spaniards tended to have the latest dinner (10 pm) followed by Italians (9 pm), French (8 pm), Germans (7 pm), and Swedes (6 pm). Thus, defining later timing using the same time criteria for all countries, or all geographical area subgroups, would be unlikely to decrease the heterogeneity across the studies. Moreover, substantial inconsistencies existed between the dietary methods and the outcome measures used in previous studies.

Different findings were reported in terms of the relationship between later timing of food intake and adiposity compared with studies combining adults and children. In their recent review, Lopez-Minguez et al^98^ suggested that a late evening meal or eating late at night increased the risk of being obese in adults, and that the risk was as high as 5 times greater, especially in evening chronotypes. However, their meta-analysis did not suggest a detrimental effect from late evening meal/eating later at night on adiposity in children/adolescents.

Similarly to previous reviews, definitive conclusions could not be drawn about the relationship between evening EI and adiposity due to the variety of evening eating occasions that the studies focused on, and the presence of conflicting results, even using the same exposure.

Previous reviews did not differentiate between evening meal, evening snack, late NE occasion (around bedtime), and total evening EI, but simply put all studies into 1 “evening EI” exposure regardless of the variety of different eating occasions across studies. This raised challenges, because the EI at different time points (ie, later at night) alone might be more relevant to adiposity than the broader “evening EI.” For this review, EI for different eating occasions as well as total evening EI were considered. No strong associations between smaller evening meal and adiposity were found, because approximately half of the studies (5/9) in this review did not show associations. Similarly, the review by Fong et al,^39^ the meta-analysis of 5 observational studies (2 studies focused on dinner, 2 on total evening EI, and 1 on late evening) did not report strong association between smaller evening EI and adiposity in adults. On the contrary, the majority of clinical trials reported that a smaller evening meal resulted in greater weight loss in adults, although their meta-analysis of 5 trials did not show differences between groups as the largest trial showed a strong reverse association between larger evening meal and adiposity. There were inconsistencies in the relationships between EI for evening snack consumption and adiposity across the studies in this review. However, a positive association between higher EI later in the evening (around bedtime) and adiposity was suggested.

The review by Almoosawi et al^41^ reported that TDEI was associated with weight status, rather than its circadian distribution. By contrast, Fong et al^39^ noted in their review that it is not likely that omitting the adjustment for TDEI affected the result, based on the comparison between adjusted and unadjusted results. Similarly, in the current review, the meta-analysis based on 4 studies suggested a positive association between higher EI at extremely late timing and adiposity; 2 out of the 4 studies adjusted for TDEI and the positive association remained. Thus, it is unlikely that omitting adjustment for TDEI affected the results in the current review. Besides, Almoosawi et al^41^ noted that it is likely that the use of absolute EI rather than proportion of TEI masked the association between time-of-day of EI and BMI. However, of the 7 studies that investigated the association between EI for evening meal and adiposity, 4 studies did use proportion of TEI for evening meal, but only 1 study showed a weak correlation. Thus, using proportion of TEI rather than absolute EI did not make a difference to the results.

### Strengths and limitations of this review

The main strength of this review is the inclusion of different definitions of NE. Later eating rhythm was analyzed comprehensively, considering 3 aspects: timing, EI, and meal frequency, and this review differentiated between EI for different eating occasions as well as total evening EI. This is the first study to review this issue in children and adolescents. Moreover, the methods were in accordance with the published protocol,^46^ making this study less likely to miss eligible studies, thus avoiding bias. The quality assessment tool for the cross-sectional studies was adapted from the Newcastle–Ottawa scale used in previous relevant studies,^48^^,^^49^ which increased the reliability and validity of the quality assessment. Finally, the GRADE system was used to rate the certainty of the evidence.

This review did have some limitations. First, it was not possible to conduct meta-analyses for all exposure variables, and not all studies were eligible to be included in the main analyses due to insufficient data or inconsistent outcomes reported across studies; when this situation arose, narrative summaries were considered instead, and results from all studies were included in the harvest plot. However, the authors are more confident in the results from the meta-analyses when both meta-analysis results and narrative synthesis results were available, because they accounted for the quality variation and heterogeneity across studies.

Second, high levels of heterogeneity were seen among the studies in the meta-analyses of exposures related to eating at later timing and evening snack consumption. This was despite the current review using strict eligibility criteria for inclusion, such as similar exposure, outcome (overweight/obesity), and statistical estimates, to ensure consistency across studies. In the meta-analysis on the relationship between eating at later timing and adiposity, it was difficult to decrease the high level of heterogeneity by defining later timing using the same time criteria for all countries (due to geographical and cultural differences in eating habits across the countries). It was not possible to perform any subgroup analysis by geographical area to overcome this, and instead the authors reported the findings via a world map and a narrative description. Apart from the inconsistent definition of exposure, the heterogeneity can also be attributed to the variability in age, sex, study design, dietary measurement, and criteria for overweight/obesity. Unfortunately, it was not possible to perform subgroup analyses due to an insufficient number of studies. However, the level of the associations by age and gender were narratively described when possible.

Third, the power of most studies was limited due to the use of less reliable or less comparable measurements for the exposure/outcome. In terms of dietary measurement, in order to capture customary or habitual eating frequency, it is important to have multiple days of data collection. However, very few studies (5/47) met this criteria. In view of the amount of information that the other 42 studies provided, this review chose not to exclude them; rather, the reliability of the dietary assessment was considered when performing quality assessments, and this was indicated by the height of each bar in the harvest plot. In addition, the level of associations of the studies using more reliable dietary assessment were compared with that of studies using any other dietary assessments, and the findings were similar (see Appendix S3 in the Supporting Information online). A considerable number of studies based their definition of overweight/obesity on their national growth charts. Studies would be more comparable if generally accepted international criteria were applied, such as IOTF or WHO cut-offs.

In addition, although no substantial differences were seen between adjusted and unadjusted results for any of the 3 aspects of later eating rhythm, it was unlikely to be able to find meaningful differences within all exposure groups because the number of studies with adjustments was insufficient. In addition, the key confounders in the relationship between later eating rhythm and adiposity still need to be determined, due to the inconsistency in the choice of confounders between studies that undertook adjusted analyses. The most common adjustments were for age, sex, ethnicity, socio-economic status, and/or physical activity. Other confounding factors (such as TEI, baseline BMI, other meal irregularity such as breakfast skipping, sleeping quality, and parental eating behavior) are likely to be relevant according to previous studies,^25^^,^^43^^,^^67^^,^^99–101^ though they are not consistently recognized as confounders in the studies included in the review. The results of this review did not differ substantially between the adjusted and unadjusted analyses. However, in 2 of the cross-sectional studies,^71^^,^^76^ subgroup analysis by physical activity level and breakfast skipping showed positive associations between EI for evening meal and BMI in children with a low physical activity level (β = 0.11, *P = .*019) and also in breakfast skippers (no estimates provided); no association between EI for evening meal and BMI were found in the group with a high level of physical activity (β = −0.083, *P = .*081) or breakfast eaters. There may also be other potential confounders that could moderate the association between NE and adiposity, such as physical activity pattern,^102^ macronutrients intake,^36^ and bedtime.^37^

Finally, the strength of evidence is already low due to the observational design of all included studies, and due to their being few cohort studies. It was also not possible to establish any causal relationship between later eating rhythm and adiposity in children/adolescents. The certainty of the evidence was downgraded to very low, mainly due to the lack of high-quality studies, inconsistency in the definitions and assessments of exposures, and also the lack of adjustment for confounders.

### Recommendations

Based on the findings of this review, there are a number of considerations for future research in this area. In terms of recommended exposures, the current studies are limited by the lack of consensus on the time criteria of “later timing” and the definition of “NE.” It is unlikely to be helpful to define a dichotomy based on a time (ie, 7 pm, 8 pm, or 10 pm) for all countries, as the beginning of biological night may differ between individuals and countries. Instead, a practical way to approximate “circadian” timing is to link the time criteria to bedtime; to date “2 hours before bedtime” is used for “later timing” most frequently in the relevant studies^98^. Given the findings of this review, future studies should focus on a combination of the timing of food consumption and evening EI. Also, studies investigating the frequency of eating occasions are urgently needed, as only 1 such study was found; the remaining studies in the meal frequency category only considered evening meal skipping or evening snack consumption. Longitudinal studies and, if possible, trials are warranted to estimate the causal relationship between later eating rhythm and adiposity in childhood/adolescence. As the findings of this review suggested, particularly for evening EI and snack consumption, there may be differences between children and adolescents, so these 2 age groups should be studied separately. To capture customary or habitual eating frequency, it is necessary to have multiple days of data collection, such as 3-day food recall/records. It is important to collect data on a wide range of relevant confounders so that they can be adjusted for in analyses. Finally, explicit guidelines in terms of energy distribution across the day in children and adolescents are needed. In the United Kingdom, Change4life set a 400–600–600 calories recommendation^103^ distributed over 3 main meals a day, for maintaining healthy weight, but did not provide more details. Similar guidelines have not been found in other countries.

## CONCLUSION

In conclusion, this review included mixed studies focusing on the relationships between different aspects of later eating rhythm and adiposity. Overall, the magnitude of the relationship between later eating rhythm and adiposity is very small. Given the distinction between exposures, analyses were conducted within each subset. Positive associations were found in meta-analyses in 2 exposure groups (higher EI around bedtime, and skipping evening main meal), but not in the other exposure subsets (such as timing of food intake, EI during different eating occasions in the evening/night, or evening snack consumption), which challenges the popular belief that “nighttime eating or higher EI in the evening is bad for weight management.”^44^ However, the findings of this review are of very low certainty due to the low quality of, and inconsistency across, studies. Further research with a more consistent definition of “later timing”, and longitudinal studies in different populations, may lead to different conclusions. The findings of this review are unlikely to be sufficient for recommending encouraging regular consumption of an evening main meal and reduction of eating around bedtime. However, the authors believe that further understanding of the effect of later eating rhythm on difficulty maintaining healthy weight in children and adolescents is important for prevention of adulthood obesity and associated chronic diseases.

## Supplementary Material

nuab079_Supplementary_Data

## Supporting Information

The following Supporting Information is available through the online version of this article at the publisher’s website.

*Figure S1*  **Study design of studies included in each exposure group**

*Figure S2*  **Sensitivity analysis for the relationship between eating at later timing (after 8 pm in general) and adiposity, after dropping low-quality studies (those scoring less than 4)**

*Figure S3*  **Meta-analysis (of 6 unique studies) on the relationship between eating at later timing (after 8 pm in general) and adiposity, using unadjusted odds ratio (OR) only**

*Figure S4*  **Meta-analysis (of 5 unique studies) on the relationship between evening meal skipping and adiposity, using unadjusted odds ratio (OR) only**

*Figure S5*  **Meta-analysis (of 7 unique studies) on the relationship between evening meal skipping and adiposity, using adjusted odds ratio (OR) only**

*Figure S6*  **Meta-analysis (of 7 unique studies) on the relationship between eating at later timing (after 8 pm in general) and adiposity, using fixed-effect model**

*Figure S7*  **Meta-analysis (of 4 unique studies) on the relationship between higher energy intake at later timing and adiposity, using random-effect model **

*Figure S8*  **Meta-analysis (of 6 unique studies) on the relationship between evening meal skipping and adiposity, using random-effect model**

*Figure S9*  **Meta-analysis (of 5 unique studies) on the relationship between evening snack consumption and adiposity, using fixed-effect model**

*Figure S10*  **Funnel plots for Egger’s test for each main meta-analysis. (a–d refer to later timing, higher energy intake at later timing, evening meal skipping, and evening snack consumption, respectively)**

*Table S1*  **Reasons for exclusion from current review**

*Table S2*  **Adapted quality assessment form**

*Table S3*  **Reasons for exclusion from meta-analysis**

*Table S4*  **The certainty of evidence according to GRADE**

*Table S5*  **Summary of results with adjustments for confounders within each exposure group**

*Appendix S1*  **Medline search strategy**

*Appendix S2*  **PRISMA checklist**

*Appendix S3*  **Sensitivity analysis after excluding studies using less reliable dietary measurements **
