# Supplementary material for: The association between later eating rhythm and adiposity in children and adolescents: a systematic review and meta-analysis
Source: Nutr Rev. 2022 May 4;80(6):1459–79. doi: 10.1093/nutrit/nuab079 (PMC9086801; doi:10.1093/nutrit/nuab079)
Supplement: nuab079_Supplementary_Data [file nuab079_supplementary_data.zip › Zou_Funnel plots for Egger test of each main meta-analysis_figure S10.docx]

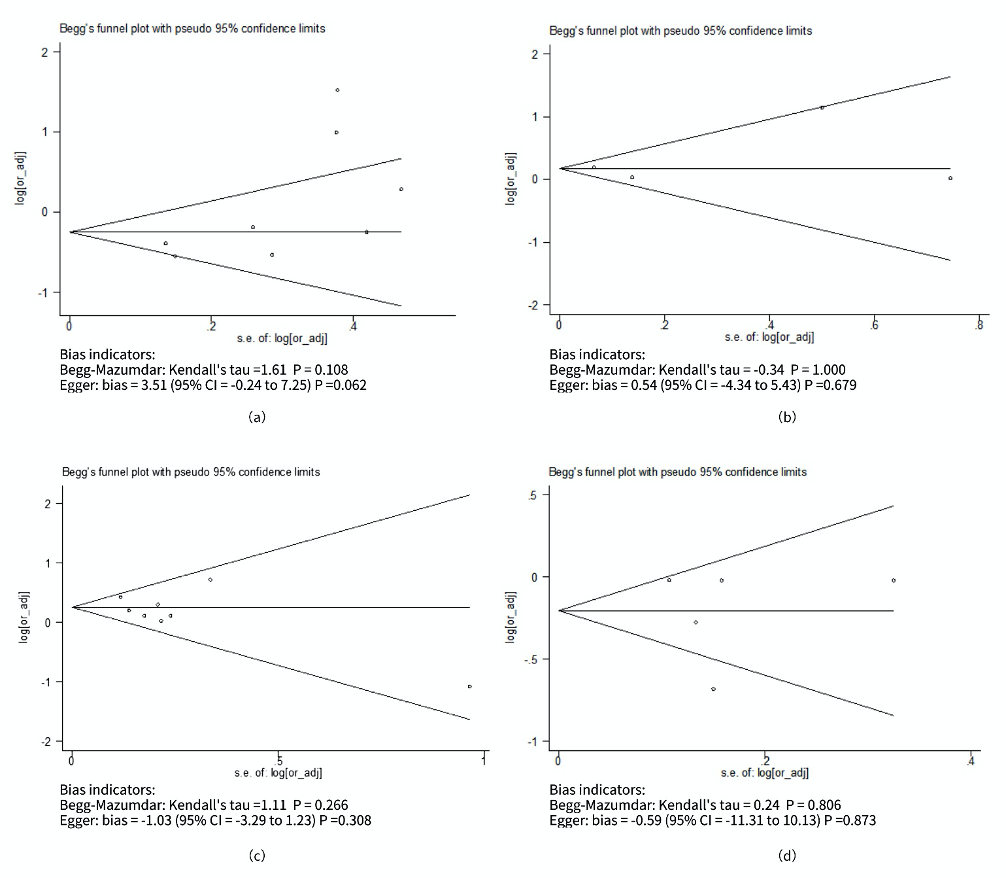


**Figure S10 Funnel plots for Egger’s test of each main meta-analysis. (a-d refer to later timing, higher energy intake at later timing, evening meal skipping and evening snack consumption, respectively).**
