# Supplementary material for: The association between later eating rhythm and adiposity in children and adolescents: a systematic review and meta-analysis
Source: Nutr Rev. 2022 May 4;80(6):1459–79. doi: 10.1093/nutrit/nuab079 (PMC9086801; doi:10.1093/nutrit/nuab079)
Supplement: nuab079_Supplementary_Data [file nuab079_supplementary_data.zip › Zou_Meta_analysis using fixed effect models_figure S6.docx]

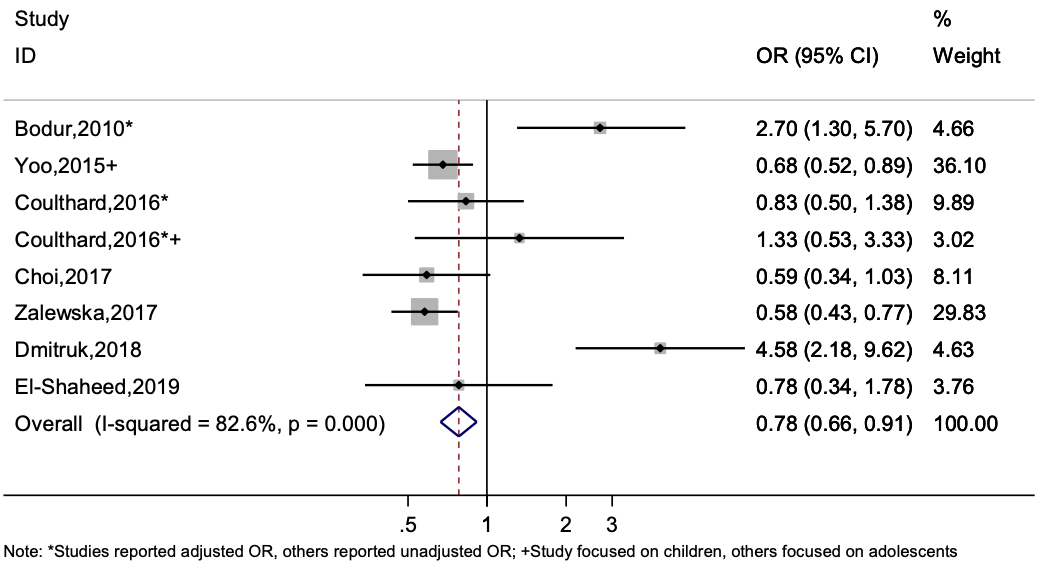


**Figure S6 Meta-analysis (7 unique studies) on the relationship between eating at later timing (after 8pm in general) and adiposity using fixed effect models.**
