# Supplementary material for: The association between later eating rhythm and adiposity in children and adolescents: a systematic review and meta-analysis
Source: Nutr Rev. 2022 May 4;80(6):1459–79. doi: 10.1093/nutrit/nuab079 (PMC9086801; doi:10.1093/nutrit/nuab079)
Supplement: nuab079_Supplementary_Data [file nuab079_supplementary_data.zip › Zou_Meta-analysis using adjusted OR only_figure S5.docx]

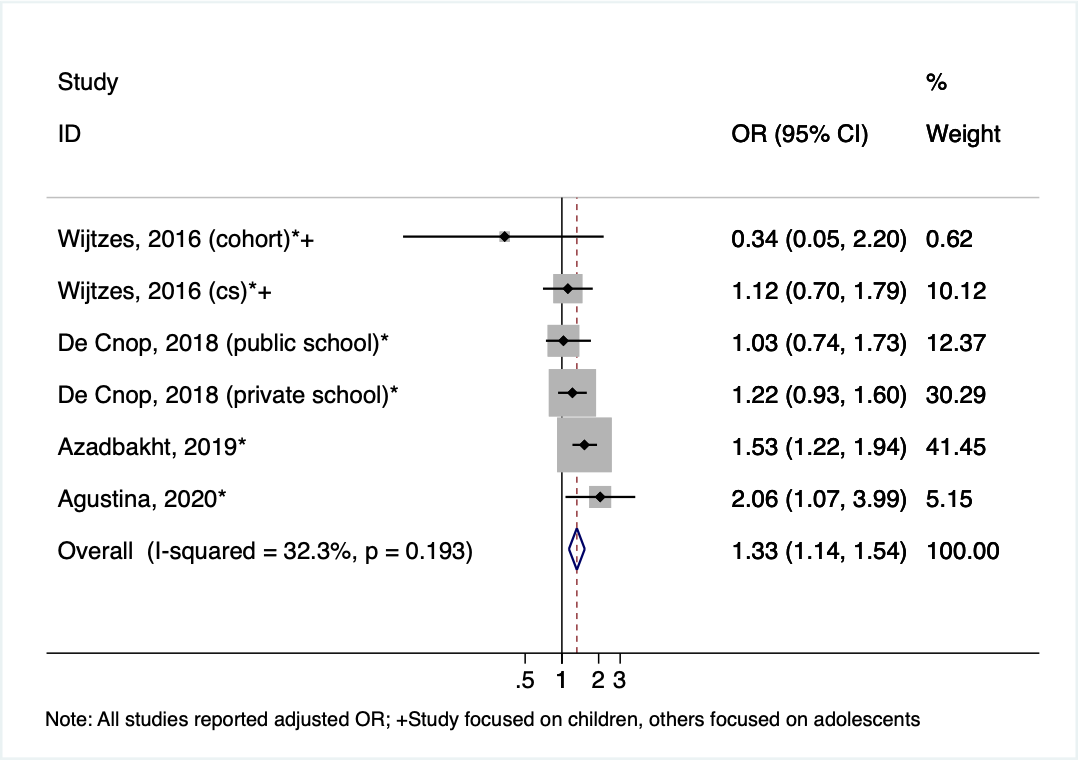


**Figure S5 Meta-analysis (7 unique studies) on the relationship between evening meal skipping and adiposity using adjusted OR only.**
