# Supplementary material for: The association between later eating rhythm and adiposity in children and adolescents: a systematic review and meta-analysis
Source: Nutr Rev. 2022 May 4;80(6):1459–79. doi: 10.1093/nutrit/nuab079 (PMC9086801; doi:10.1093/nutrit/nuab079)
Supplement: nuab079_Supplementary_Data [file nuab079_supplementary_data.zip › Zou_Quality assessment form_table S2.docx]

**Table S2 Adapted quality assessment form**

**Risk of bias assessment**

## The modified Newcastle Ottawa scale for cross sectional studies

| Categories and Items | **Newcastle-Ottawa Scale** | *Number stars* |
| --- | --- | --- |
| ***Selection (Maximum 5 stars)*** |  |  |
| 1. Representativeness of the sample | a) Truly representative of the average in the target population. * (all subjects or random sampling)  b) Somewhat representative of the average in the target population. * (nonrandom sampling)  c) Selected group of users.  d) No description of the sampling strategy. |  |
| 1. Sample size | a) Justified and satisfactory. *  b) Not justified. |  |
| 1. Non-respondents | a) Comparability between respondents and non-respondents characteristics is established, and the response rate is satisfactory. *  b) The response rate is unsatisfactory, or the comparability between respondents and non-respondents is unsatisfactory.  c) No description of the response rate or the characteristics of the responders and the non-responders |  |
| 1. Ascertainment of the exposure (risk factor) | a) Validated measurement tool. **  b) Non-validated measurement tool, but the tool is available or described.*  c) No description of the measurement tool. |  |
| ***Comparability (Maximum 2 stars)*** |  |  |
| 1. The subjects in different outcome groups are comparable, based on the study design or analysis. Confounding factors are controlled | a) The study controls for the most important factor (sedentary behaviour). **  b) The study control for any additional factor. * |  |
| ***Outcome (Maximum 3 stars)*** |  |  |
| 1. Assessment of the outcome | a) Independent blind assessment. **  b) Record linkage. **  c) Self report.  d) No description. |  |
| 1. Statistical test | a) The statistical test used to analyze the data is clearly described and appropriate, and the measurement of the association is presented, including confidence intervals and the probability level (p value). *  b) The statistical test is not appropriate, not described or incomplete. |  |
| Total stars: | | |
| *This scale has been adapted from the Newcastle-Ottawa Quality Assessment Scale for cohort studies to perform a quality assessment of cross-sectional studies for the systematic review | | |

## b. Newcastle-Ottawa Scale for assessing cohort and case control studies

| Categories and Items | **Newcastle-Ottawa Scale** | *Number stars* |
| --- | --- | --- |
| ***Selection*** |  |  |
| Is the exposed cohort representative of the general population? | 1. Truly representative of the average. (1 star) 2. Somewhat representative of the average (1 star) 3. Selected groups (ex. Nurses, volunteers) 4. No description of the derivation of the cohort. |  |
| Is the non-exposed cohort somewhat representative of the general population?  *With these item we wonder whether the sample is representative of the general population. In my view, we should give more value to all those cohort studies that used representative samples of the population. Example, if we only obtained cohorts of high social class individuals residual confounding may occurs and therefore we can obtain invalid conclusions***.** | 1. Drawn from the same community as the exposed cohort. (1 star) 2. Drawn from a different source. 3. No description. |  |
| Exposure measurement  By validated tools or structured interview | 1. Structured interview (1 star) 2. Secure record (1 star) (example, weighting food, taking photos) 3. Self-reports 4. No description |  |
| Demonstration that the outcome of interest was not present at start of study  In the case of mortality studies, outcome of interest is still the presence of a disease/incident, rather than death. That is to say that a statement of no history of disease or incident earns a star. | 1. Yes (1 star) 2. No |  |
| **Comparability** | Models adjusted by age? Yes No | If Yes 1 star |
|  | By age + or under-over reporting energy intake?  Yes No | If Yes 1 star |
| **Outcome** | In a laboratory by standardized methods (body composition and diabetes) or medical records (independent blind assessment or record linkage) for CVD Yes No | If Yes 1 star |
|  | Follow-up enough for outcomes to occur? Yes No (possibly base on more or less than 1 year or follow-up) | If Yes 1 star |
|  | Subjects lost to follow up unlikely to produce bias (> 90% completion rate)  Yes No | If Yes 1 star |
| TOTAL STARS | | |
