# Supplementary material for: The association between later eating rhythm and adiposity in children and adolescents: a systematic review and meta-analysis
Source: Nutr Rev. 2022 May 4;80(6):1459–79. doi: 10.1093/nutrit/nuab079 (PMC9086801; doi:10.1093/nutrit/nuab079)
Supplement: nuab079_Supplementary_Data [file nuab079_supplementary_data.zip › Zou_Reasons for exclusion from meta-analysis_table S3.docx]

| **Table S3 Reasons for exclusion from meta-analysis** | | | | | |
| --- | --- | --- | --- | --- | --- |
| **Exposure** | **Outcome** | **Study** | **Study design** | **Exclusion Reason** | |
|  |  |  |  | **Sufficient data (OR)  for meta-analysis** | **Sufficient studies (n>3) in each subset** |
| **Eating at later timing** | Overweight | Watanabe et al. (2016)^S1^ | CS | **No- P value only** | **No- n=0** |
|  | Obesity | Alavi Naini et al. (2006)^S2^ | CC | Yes- OR 0.70 (0.27 to 1.80) | **No- n=2** |
|  |  | Ayine et al. (2018)^S3^ | Abstract | **No- Abstract** |  |
|  |  | Rychkova et al. (2019)^S4^ | CC | Yes- OR 1.66 (0.89 to 3.12) |  |
|  | Overweight/obesity | Barbu et al. (2015)^S5^ | CS | **No- no data** | Yes- n=7 |
|  | BMI Z-score | Huang et al. (2014)^S6^ | Abstract-CS | **No- Abstract** | **No- n=0** |
| **EI for evening main meal** | Obesity | Waxman & Stunkard (1980)^S7^ | CC | **No- F [1, 33] =23.42, P<0.001** | **No- n=0** |
|  | Overweight/obesity | Azizi F et al. (2001)^S8^ | CS | **No- P value only** | **No- n=1** |
|  |  | Eloranta et al. (2012)^S9^ | CS | Yes- OR 0.96 (0.92 to 1.00) |  |
|  |  | Vilela et al. (2019)^S10^ | Cohort study | **No- P value only** |  |
|  | BMI/BMI Z-score | Dubois et al. (2009)^S11^ | CS | **No- no data** | **No- n=0** |
|  |  | Fayet et al. (2012)^S12^ | CS | **No- no data** |  |
|  |  | Karatzi et al. (2017)^S13^ | CS | **No- β=0.03, P=0.214** |  |
|  |  | Mayorga Mazon et al. (2017)^S14^ | Abstract-CS | **No- Abstract** |  |
|  | Other | Maffeis et al. (2000)^S15^ | CS | **No- r=0.10 P<0.05** | **No- n=0** |
| **EI for evening snack** | Overweight/obesity | Azizi F et al. (2001)^S8^ | CS | **No- P value only** | **No- n=1** |
|  |  | Bo et al. (2014)^S16^ | CS | Yes- OR 3.12 (1.17 to 8.34) |  |
|  |  | Vilela et al. (2019)^S10^ | Cohort study | **No- P value only** |  |
|  | BMI/BMI Z-score | Fayet et al. (2012)^S12^ | CS | **No- no data** | **No- n=0** |
|  |  | Karatzi et al. (2017)^S13^ | CS | **No- β=0.004, P=0.878** |  |
|  | Other | Maffeis et al. (2000)^S15^ | CS | **No- r=-0.17 P<0.001** | **No- n=0** |
| **EI for whole evening** | Overweight | Eng et al. (2009)^S17^ | CS | **No- β=16.70, P=0.007; β=-15.90, P=0.009** | **No- n=0** |
|  | Obesity | Eng et al. (2009)^S17^ | CS | **No- β=10.30, P=0.184; β=-5.20, P=0.518** | **No- n=0** |
|  | BMI/BMI Z-score | Thompson et al. (2006)^S18^ | Cohort study | **No- β=1.41, P=0.039** | **No- n=0** |
| **EI around bedtime** | Overweight | Hernandez et al. (2016)^S19^ | CS | Yes- OR 0.90 (0.54 to 1.40) | **No- n=1** |
|  | Obesity | Hernandez et al. (2016)^S19^ | CS | Yes- 1.13 (0.65 to 1.98) | **No- n=1** |
|  | BMI/BMI Z-score | Hernandez et al. (2016)^S19^ | CS | **No- β=0.18, P=0.007** | **No- n=0** |
|  |  | Yüksel et al. (2017)^S20^ | CS | **No- P value only** |  |
| **Evening main meal skipping** | Overweight | Lioret et al. (2008)^S21^ | CS | Yes- OR 0.89 (0.46 to 1.70) | **No- n=1** |
|  | Overweight/obesity | Reed et al. (2013)^S22^ | CS | **No- P value only** | Yes- n=5 |
|  |  | Vik et al. (2013)^S23^ | CS | **No- P value only** |  |
|  | BMI/BMI Z-score | Lehto et al. (2011)^S24^ | CS | **No- B=-0.45 (-1.1 to 0.15)** | **No- n=1** |
|  |  | Ostachowska-Gasior (2016)^S25^ | CS | Yes- OR 1.03 (0.99 to 1.09) |  |
|  |  | Yorulmaz et al. (2012)^S26^ | CS | **No- P value only** |  |
|  | Other | Gomez-Martin et al. (2012)^S27^ | CS | **No- P value only** | **No- n=0** |
|  |  | Taib et al. (2014)^S28^ | Abstract-CS | **No- Abstract** |  |
| **Table S3 Continued** | | | | | |
| **Exposure** | **Outcome** | **Study** | **Study design** | **Exclusion Reason** | |
|  |  |  |  | **Sufficient data (OR)  for meta-analysis** | **Sufficient studies (n>3) in each subset** |
| **Evening snack consumption** | Obesity | Ben Slama et al. (2002)^S29^ | CC | Yes- OR 7.97 (4.19 to 15.15) | **No- n=2** |
|  |  | Rychkova et al. (2019)^S4^ | CC | Yes- OR 1.16 (0.40 to 3.37) |  |
|  | Overweight/obesity | Cezimbra et al. (2019)^S30^ | Abstract-CS | **No- Abstract** | Yes- n=5 |
|  | Other | Gomez-Martin et al. (2012)^S27^ | CS | **No- P value only** | **No- n=0** |
| * Other outcome refer to one of other markers of adiposity: waist circumference; skinfold; fat mass/ %fat.  Abbreviations: OR, odds ratio; CS, cross-sectional study; CC, case control study; BMI, body mass index; EI, energy intake. | | | | | |

**Supplementary References**

1. Watanabe E, Lee JS, Mori K, Kawakubo K. Clustering patterns of obesity-related multiple lifestyle behaviours and their associations with overweight and family environments: a cross-sectional study in Japanese preschool children. BMJ open, 2016;6(11).
2. Alavi NA, Amini M, Karajibani M, et al. Association of obesity with food habits and body image in school children of Nakhon Pathom Province, Thailand, 2006.
3. Ayine P, Parra EP, Jeganathan RB, Thangiah G. Influence of Race, Ethnicity, and Behavioral Factors on Childhood Obesity. 2018.
4. Rychkova L, Pogodina A, Ayurova Z, Berdina O. Risk Factors for Obesity in Adolescents Living in Rural Areas of Buryatia: A Case-Control Study. J Biomed, 2019;9(2): pp.190-195.
5. Barbu CG, Teleman MD, Albu AI, et al. Obesity and eating behaviors in school children and adolescents–data from a cross sectional study from Bucharest, Romania. BMC Public Health, 2015;15(1): p.206.
6. Huang Y, Ho SY, Huang R, Lo WS, Lam TH. Night eating in Hong Kong adolescents: prevalence and associations with dinner habits, bedtime and weight status. The University of Hong Kong (Pokfulam, Hong Kong), 2014.
7. Waxman M, Stunkard AJ. Caloric intake and expenditure of obese boys. J Pediatr, 1980;96(2): pp.187-193.
8. Azizi F, Allahverdian S, Mirmiran P, Rahmani M, Mohammadi F. Dietary factors and body mass index in a group of Iranian adolescents: Tehran lipid and glucose study-2. Int J Vitam Nutr Res, 2001;71(2): pp.123-127.
9. Eloranta AM, Lindi V, Schwab U, et al. Dietary factors associated with overweight and body adiposity in Finnish children aged 6–8 years: the PANIC Study. Int J Obes (Lond), 2012;36(7): pp.950-955.
10. Vilela S, Oliveira A, Severo M, Lopes C. Chrono-Nutrition: The Relationship between Time-of-Day Energy and Macronutrient Intake and Children’s Body Weight Status. J Biol Rhythms, 2019;34(3): pp.332-342.
11. Dubois L, Girard M, Kent MP, Farmer A, Tatone-Tokuda F. Breakfast skipping is associated with differences in meal patterns, macronutrient intakes and overweight among pre-school children. Public Health Nutr, 2009;12(1): pp.19-28.
12. Fayet F, Mortensen A, Baghurst K. Energy distribution patterns in Australia and its relationship to age, gender and body mass index among children and adults. Nutr Diet, 2012;69(2): pp.102-110.
13. Karatzi K, Moschonis G, Choupi E, et al. Late-night overeating is associated with smaller breakfast, breakfast skipping, and obesity in children: The Healthy Growth Study. Nutrition, 2017;33: pp.141-144.
14. Mayorga Mazon CDLM, Monzon Rodriguez AN, Ligerini Vazquez LJ, Menendez Blanco CY, Guerendiain Margni ME. Energy, Protein and carbohydrate intake in relation to anthropometric parameters at different meal times in children of evanes study. Ann Nutr Metab, 2017;71: pp. 609-609.
15. Maffeis C, Provera S, Filippi L, et al. Distribution of food intake as a risk factor for childhood obesity. Int J Obes (Lond), 2000;24(1): pp.75-80.
16. Bo S, De Carli L, Venco E, et al. Impact of snacking pattern on overweight and obesity risk in a cohort of 11-to 13-year-old adolescents. J Pediatr Gastroenterol Nutr, 2014;59(4), pp.465-471.
17. Eng S, Wagstaff DA, Kranz S. Eating late in the evening is associated with childhood obesity in some age groups but not in all children: the relationship between time of consumption and body weight status in US children. Int J Behav Nutr Phy, 2009;6(1): p.27.
18. Thompson OM, Ballew C, Resnicow K, et al. Dietary pattern as a predictor of change in BMI z-score among girls. Int J Obes (Lond), 2006;30(1): pp.176-182.
19. Hernandez E, Kim M, Kim WG, Yoon J. Nutritional aspects of night eating and its association with weight status among Korean adolescents. Nutr Res Pract, 2016;10(4): pp.448-455.
20. Yüksel A, Önal HY, Kurt KG. Adherence to the Mediterranean diet and factors affecting obesity in high school students. Int J Med Sci Public Health, 2017;6(12): pp.78-86.
21. Lioret S, Touvier M, Lafay L, Volatier JL, Maire B. Are eating occasions and their energy content related to child overweight and socioeconomic status?. Obes, 2008;16(11): pp.2518-2523.
22. Reed M, Dancy B, Holm K, Wilbur J, Fogg L. Eating behaviors among early adolescent African American girls and their mothers. J Sch Nurs, 2013;29(6): pp.452-463.
23. Vik FN, Bjørnarå HB, Øverby NC, et al. Associations between eating meals, watching TV while eating meals and weight status among children, ages 10–12 years in eight European countries: the ENERGY cross-sectional study. Int J Behav Nutr Phys Act, 2013;10(1): p.58.
24. Lehto R, Ray C, Lahti-Koski M, Roos E. Meal pattern and BMI in 9–11-year-old children in Finland. Public Health Nutr, 2011;14(7), pp.1245-1250.
25. Ostachowska-Gasior A, Piwowar M, Kwiatkowski J, Kasperczyk J, Skop-Lewandowska A. Breakfast and other meal consumption in adolescents from southern Poland. Int J Environ Res Public Health, 2016;13(5): p.453.
26. Yorulmaz H, Pacal FP. Assessment of Nutritional Habits and Obesity Situations of Adolescents in 16-18 Age Group. Turkiye Klinikleri Tip Bilimleri Dergisi. 2012;32(2):364-370.
27. Gómez-Martínez S, Martínez-Gómez D, de Heredia FP, et al. Eating habits and total and abdominal fat in Spanish adolescents: influence of physical activity. The AVENA study. J Adolesc Health, 2012;50(4): pp.403-409.
28. Taib M, Chin Y, Wahida F, Kaartina S, Woon F, Zalilah M. Meal skipping as a risk factor of abdominal obesity among Malaysian adolescents: findings from the Malaysian overweight and disordered eating survey: T3: S14. 35. Obes Rev, 2014;15.
29. Ben Slama F, Achour A, Belhadj O, Hsairi M, Oueslati M, Achour N. Obesity and way of life in a schoolboy population of the Ariana region (Tunisia) aged of 6 to 10 years. Tunis Med, 2002;80(9): pp.542-547.
30. Cezimbra VG, De Oliveira MT, Pereira LJ, et al. Meal intake and overweight in schoolchildren aged 7 to 12 years old in a city in southern brazil. Obes Facts, 2019;12 (Supplement 1): p. 207.
