# Supplementary material for: The association between later eating rhythm and adiposity in children and adolescents: a systematic review and meta-analysis
Source: Nutr Rev. 2022 May 4;80(6):1459–79. doi: 10.1093/nutrit/nuab079 (PMC9086801; doi:10.1093/nutrit/nuab079)
Supplement: nuab079_Supplementary_Data [file nuab079_supplementary_data.zip › Zou_Reasons for exclusion from the current review_table S1.docx]

| **Table S1 Reasons for exclusion from the current review** | | | | | | | |
| --- | --- | --- | --- | --- | --- | --- | --- |
| **No** | **Author (Year)** | **M ZOU** | **Reason for exclusion** | **Kate N** | **Reason for exclusion** | **Final decision** | **Final reason for exclusion** |
| S1 | Aljuraiban (2015) | excluded | the population are adult aged from 40 to 59 | N | Adults | excluded | Not a population of interest |
| S2 | Alnaim (2017) | excluded | 24 months | Y | aged 24 months | excluded | Not a population of interest |
| S3 | Ayine (2018) | N/A | abstract | Y | but no TDEI | included |  |
| S4 | Barbu (2015) | included |  | Y | but no TDEI | included |  |
| S5 | Baron (2013) | excluded | the population are adult aged from 18-71 | N | adults | excluded | Not a population of interest |
| S6 | Baron (2011) | excluded | the population are adult aged from 18-72 | N | adults | excluded | Not a population of interest |
| S7 | Choi (2017) | included |  | Y | but no TDEI | included |  |
| S8 | Coulthard (2016) | included |  | Y |  | included |  |
| S9 | Dmitruk (2018) | included | girls only | Y | but no TDEI | included |  |
| S10 | Eng (2009) | included |  | Y |  | included |  |
| S11 | Fayet (2012) | included |  | Y |  | included |  |
| S12 | Fisher (2007) | included |  | N | Experimental - about eating when not hungry | excluded | Not an exposure of interest |
| S13 | Hernanderz (2016) | included |  | Y |  | included |  |
| S14 | Karatzi (2017) | included |  | Y |  | included |  |
| S15 | Lamerz (2005) | included |  | Y |  | included |  |
| S16 | Maffeis (2000) | included |  | Y |  | included |  |
| S17 | Mayorga (2017) | included | abstract | N | Effects of fortification (focus on lunchtimes) | included |  |
| S18 | MacHill (2017) | excluded | the population are young adult aged 18-22 | N | adults | excluded | Not a population of interest |
| **Table S1 continued** | | | | | | | |
| S19 | Meule (2014) | excluded | the population are adult aged from 21-70+ | N | Adults | excluded | Not a population of interest |
| S20 | Striegel-Moore (2006) | excluded | all age | Y | all age group | excluded | Not a population of interest |
| S21 | Striegel-Moore (2008) | excluded | the age group is 15-39 years, and no subgroup of it | N | children not exmained separately, so cannot pull out data | excluded | Not a population of interest |
| S22 | Thompson (2006) | included |  | Y |  | included |  |
| S23 | Yuksel (2017) | included |  | Y | but no TDEI | included |  |
| S24 | Zalewska (2017) | included |  | Y | but no TDEI | included |  |
| S25 | Ahmed (2013) | excluded | did not mention evening/night food intake, fruit consumption only | N | No night eating | excluded | Not an exposure of interest |
| S26 | Abbes (2016) | excluded | non-English language, French | N | Not in English but abstract suggests no TDEI | excluded | Not an exposure of interest |
| S27 | Ben Slama (2002) | included | non-English language, French | Y | no TDEI | included |  |
| S28 | Berkowitz (2010) | excluded | did not mention evening/night food intake, TEI only | N | but no TDEI | excluded | Not an exposure of interest |
| S29 | Bo (2014) | included |  | Y | but no TDEI | included |  |
| S30 | Cassani (2015) | excluded | abstract | N | adults (+ I don't really understand the paper!) | excluded | Not a population of interest |
| S31 | Cole (2016) | excluded | abstract, not relevant to night time eating | Y | but no TDEI | excluded | Not an exposure of interest |
| S32 | De Cnop (2018) | included | dinner regulation | Y | but no TDEI | included |  |
| S33 | Kuperk (2016) | excluded | specific food | N | specific food | excluded | Not an exposure of interest |
| S34 | Lehto (2011) | included | dinner regulation | Y | but no TDEI | included |  |
| **Table S1 continued** | | | | | | | |
| S35 | Lioret (2008) | excluded | Abstract-lunch and dinner together but not specifically on dinner timing | N | Main meal contribution to TDEI only, not broken down by mealtime | excluded | Not an exposure of interest |
| S36 | Lundgren (2012) | excluded | did not associate night eating and adiposity, prevalence only | Y | but no TDEI | excluded | Not an outcome of interest |
| S37 | Meule (2014) | excluded | participant aged 18+ | N | adults, No TDEI | excluded | Not a population of interest |
| S38 | Mohd Taib (2014) | included | Abstract. dinner skipping | Y | but no TDEI | included |  |
| S39 | Moreno (2007) | excluded | review | N | Systematic review (Check refs though) | excluded | Review paper |
| S40 | Pham (2012) | excluded | review, did not mention adiposity, not relevant | N | Systematic review (Check refs though) | excluded | Review paper |
| S41 | Rodriguez (2008) | N/A | article not found | ? |  | excluded | Could not access |
| S42 | Tsiaousi (2012) | excluded | non-English language, Greek; breakfast, no the relationship between dinner and BMI | ? | Not in English | N/A | Not an outcome of interest |
| S43 | Watanabe (2016) | included |  | Y | but no TDEI | included |  |
| S44 | Waxman (1980) | included |  | ? | Peculiar study design, need to discuss | included |  |
| S45 | Wijitzes (2016) | included | dinner regulation | N | Focus on meal skipping and no TDEI | included |  |
| S46 | Wijtzes (2015) | excluded | did not mention adiposity relevant variables | N | Focus on meal skipping and no TDEI | excluded | Not an outcome of interest |
| S47 | Yeh (2014) | excluded | participant aged 18+ | N | adults, No TDEI | excluded | Not a population of interest |
| S48 | Yoo (2015) | included |  | Y | but no TDEI | included |  |
| S49 | Yorulmaz (2012) | included | non-English, Turkish | Y | but no TDEI | included |  |
| **Table S1 continued** | | | | | | | |
| S50 | Bodur (2012) | included |  | Y | but no TDEI | included |  |
| S51 | Ciccone (2013) | included | evening snack | N | all data there but not broken down in the way needed | Y |  |
| S52 | Dubois (2009) | included |  | Y |  | included |  |
| S53 | Gluck (2008) | excluded | participant aged 26+ | N | adults | excluded | Not a population of interest |
| S54 | Ostachowska-Gasior (2016) | included | dinner regulation | Y | but no TDEI | included |  |
| S55 | Prochnik Estima (2009) | excluded | investigate the main meals together but not dinner independently | Y | but no TDEI | excluded | Not an exposure of interest |
| S56 | Tee (2018) | excluded | outcomes are executive function | N | no weight status | excluded | Not an outcome of interest |
| S57 | Xiong (2008) | excluded | did not assess behaviour but attitude only | ? | I can't open the document | excluded | Not an exposure of interest |
| S58 | Azadbakht (2019) | included |  | Y |  | included |  |
| S59 | Palla and Almoosawi (2019) | excluded | eating pattern: main meal and night snack | N | diurnal eating pattern | excluded | Not an exposure of interest |
| S60 | Top (2019) | included | evening snack | Y | evening snack | included |  |
| S61 | Vilela (2019) | included | energy intake for dinner | Y | but no TDEI | included |  |
| S62 | Cezimbra (2019) | included | abstract | Y |  | included |  |
| S63 | Lioret (2008) | included | dinner skipping | Y |  | included |  |
| S64 | Musaiger (2014) | included | evening meal/snack | Y |  | included |  |
| S65 | Onyiriuka (2013) | excluded | any meal skipping | N | not focused on night | excluded | Not an exposure of interest |
| S66 | Muñoz 2016 | excluded | adult | N | Adult | excluded | Not a population of interest |
| **Table S1 continued** | | | | | | | |
| S67 | Ochiai (2013) | included | evening snack | Y | evening snack | included |  |
| S68 | Gomez-Martin (2012) | included |  | Y |  | included |  |
| S69 | Vik (2013) | included |  | Y |  | included |  |
| S70 | Reed (2012) | included |  | Y |  | included |  |
| S71 | Rychkova (2017) | included |  | Y |  | included |  |
| S72 | EI-Shaheed (2019) | included |  | Y |  | included |  |
| S73 | Alavi Naini (2006) | included |  | Y |  | included |  |
| S74 | Eloranta (2012) | included |  | Y |  | included |  |
| S75 | Azizi (2001) | included |  | Y |  | included |  |
| S76 | Huang (2014) | included | Abstract | Y | but abstract | included |  |
| S77 | Sun (2020) | included | evening snack | Y | but no TDEI | included |  |
| S78 | Agustina (2020) | included | dinner skipping | Y |  | included |  |
| S79 | Abduelkarem (2020) | excluded | no relation to outcome | Y |  | excluded | Not an outcome of interest |

**Supplementary References**

1. Aljuraiban GS, Chan Q, Oude Griep LM, et al. The Impact of Eating Frequency and Time of Intake on Nutrient Quality and Body Mass Index: The INTERMAP Study, a Population-Based Study. Journal of the Academy of Nutrition & Dietetics. 2015;115(4):528-536.e521.
2. Alnaim LA, Taylor M, Thodosoff J, Sullivan D, Carlson S. The effect of nighttime energy intake on BMI-Z scores during early childhood. FASEB Journal Conference: Experimental Biology. 2017;31(1 Supplement 1).
3. Ayine P, Parra EP, Jeganathan RB, Thangiah G. Influence of race, ethnicity, and behavioral factors on childhood obesity. Diabetes. 2018;67 (Supplement 1):A546.
4. Barbu CG, Teleman MD, Albu AI, et al. Obesity and eating behaviors in school children and adolescents -data from a cross sectional study from Bucharest, Romania. BMC public health. 2015;15:206.
5. Baron KG, Reid KJ, Horn LV, et al. Contribution of evening macronutrient intake to total caloric intake and body mass index. Appetite. 2013;60:246-251.
6. Baron KG, Reid KJ, Kern AS, et al. Role of sleep timing in caloric intake and BMI. Obesity (19307381). 2011;19(7):1374-1381.
7. Choi MK, Cho YJ, Kim MH, Bae YJ. Night eating status according to body mass index of Korean adolescents. Nutrition & Food Science. 2017;47(1):89-100.
8. Coulthard JD, Pot GK. The timing of the evening meal: how is this associated with weight status in UK children? British Journal of Nutrition. 2016;115(9):1616-1622.
9. Dmitruk A, Kunicka I, Poplawska H, Holub W. Dietary patterns among girls aged 16-18 years old according to their body mass index and waist-to-hip ratio. Pediatria I Medycyna Rodzinna-Paediatrics and Family Medicine. 2018;14(1):78-87.
10. Eng S, Wagstaff DA, Kranz S. Eating late in the evening is associated with childhood obesity in some age groups but not in all children: the relationship between time of consumption and body weight status in U.S. children. International Journal of Behavioral Nutrition & Physical Activity. 2009;6:27.
11. Fayet F, Mortensen A, Baghurst K. Energy distribution patterns in Australia and its relationship to age, gender and body mass index among children and adults. Nutrition & Dietetics. 2012;69(2):102-110.
12. Fisher JO, Arreola A, Birch LL, Rolls BJ. Portion size effects on daily energy intake in low-income Hispanic and African American children and their mothers. American Journal of Clinical Nutrition. 2007;86(6):1709-1716.
13. Hernandez E, Kim M, Kim WG, Yoon J. Nutritional aspects of night eating and its association with weight status among Korean adolescents. Nutrition Research & Practice. 2016;10(4):448-455.
14. Karatzi K, Moschonis G, Choupi E, Manios Y, Healthy Growth Study g. Late-night overeating is associated with smaller breakfast, breakfast skipping, and obesity in children: The Healthy Growth Study. Nutrition. 2017;33:141-144.
15. Lamerz A, Kuepper-Nybelen J, Bruning N, et al. Prevalence of obesity, binge eating, and night eating in a cross-sectional field survey of 6-year-old children and their parents in a German urban population. Journal of Child Psychology & Psychiatry & Allied Disciplines. 2005;46(4):385-393.
16. Maffeis C, Provera S, Filippi L, et al. Distribution of food intake as a risk factor for childhood obesity. International Journal of Obesity. 2000;24(1):75-80.
17. Mayorga Mazon CDLM, Monzon Rodriguez AN, Ligerini Vazquez LJ, Menendez Blanco CY, Guerendiain Margni ME. Energy, protein and carbohydrate intake in relation to anthropometric parameters at different mealtimes in children of evanes study. Annals of Nutrition and Metabolism. 2017;71 (Supplement 2):609.
18. McHill AW, Phillips AJK, Czeisler CA, et al. Later circadian timing of food intake is associated with increased body fat. American Journal of Clinical Nutrition. 2017;106(5):1213-1219.
19. Meule A, Allison KC, Brähler E, de Zwaan M. The association between night eating and body mass depends on age. Eating Behaviors. 2014;15(4):683-685.
20. Striegel-Moore RH, Franko DL, Thompson D, Affenito S, Kraemer HC. Night eating: prevalence and demographic correlates. Obesity. 2006;14(1):139-147.
21. Striegel-Moore RH, Franko DL, Thompson D, Affenito S, May A, Kraemer HC. Exploring the typology of night eating syndrome. International Journal of Eating Disorders. 2008;41(5):411-418.
22. Thompson OM, Ballew C, Resnicow K, et al. Dietary pattern as a predictor of change in BMI z-score among girls. International Journal of Obesity. 2006;30(1):176-182.
23. Yuksel A, Onal HY, Kurt KG. Adherence to the Mediterranean Diet and Factors Affecting Obesity in High School Students. International Journal of Medical Research & Health Sciences. 2017;6(12):78-86.
24. Zalewska M, Maciorkowska E. Selected nutritional habits of teenagers associated with overweight and obesity. PeerJ. 2017;5:e3681.
25. Ahmed J, Laghari A, Naseer M, Mehraj V. Prevalence of and factors associated with obesity among Pakistani schoolchildren: a school-based, cross-sectional study. Eastern Mediterranean Health Journal. 2013;19(3):242-247.
26. Abbes MA, Bereksi-Reguig K. Risk factors for obesity among school aged children in western Algeria: results of a study conducted on 293 subjects. Tunisie Medicale. 2016;94(1):23-28.
27. Ben Slama F, Achour A, Belhadj O, Hsairi M, Oueslati M, Achour N. Obesity and way of life in a schoolboy population of the Ariana region (Tunisia) aged of 6 to 10 years. [French]. Tunisie Medicale. 2002;80(9):542-547.
28. Berkowitz RI, Moore RH, Faith MS, Stallings VA, Kral TV, Stunkard AJ. Identification of an obese eating style in 4-year-old children born at high and low risk for obesity.[Erratum appears in Obesity (Silver Spring). 2010 Mar;18(3):648]. Obesity. 2010;18(3):505-512.
29. Bo S, De Carli L, Venco E, et al. Impact of snacking pattern on overweight and obesity risk in a cohort of 11-to 13-year-old adolescents. Journal of Pediatric Gastroenterology and Nutrition. 2014;59(4):465-471.
30. Cassani R, Amancio OM, Geloneze B, Pareja J. Lack fractionation meals: Risk for behavior changes cardiometabolic. FASEB Journal Conference: Experimental Biology. 2015;29(1 Meeting Abstracts).
31. Cole NC, Musaad SM, Fiese BH, Lee SY, Donovan SM. Influence of family mealtime routines on preschoolers' picky eating behaviors and food consumption. FASEB Journal Conference: Experimental Biology. 2016;30(Meeting Abstracts).
32. De Cnop ML, Monteiro LS, Rodrigues PRM, Estima CCP, da Veiga GV, Pereira RA. Meal habits and anthropometric indicators in adolescents from public and private schools of the metropolitan region of Rio de Janeiro. Revista De Nutricao-Brazilian Journal of Nutrition. 2018;31(1):35-47.
33. Kupek E, Lobo AS, Leal DB, Bellisle F, de Assis MA. Dietary patterns associated with overweight and obesity among Brazilian schoolchildren: an approach based on the time-of-day of eating events. British Journal of Nutrition. 2016;116(11):1954-1965.
34. Lehto R, Ray C, Lahti-Koski M, Roos E. Meal pattern and BMI in 9-11-year-old children in Finland. Public Health Nutrition. 2011;14(7):1245-1250.
35. Lioret S, Touvier M, Lafay L, Volatier JL, Maire B. Are eating occasions and their energy content related to child overweight and socioeconomic status? Obesity. 2008;16(11):2518-2523.
36. Lundgren JD, Drapeau V, Allison KC, et al. Prevalence and Familial Patterns of Night Eating in the Québec Adipose and Lifestyle InvesTigation in Youth (QUALITY) Study. Obesity (19307381). 2012;20(8):1598-1603.
37. Meule A, Allison KC, Platte P. Emotional Eating Moderates the Relationship of Night Eating with Binge Eating and Body Mass. European Eating Disorders Review. 2014;22(2):147-151.
38. Mohd Taib M, Chin Y, Fara Wahida R, Kaartina S, Woon F, Zalilah M. Meal skipping as a risk factor of abdominal obesity among Malaysian adolescents: Findings from the Malaysian overweight and disordered eating survey. Obesity Reviews. 2014;2):63.
39. Moreno LA, Rodriguez G. Dietary risk factors for development of childhood obesity. Current Opinion in Clinical Nutrition & Metabolic Care. 2007;10(3):336-341.
40. Pham DD, Lee JC, Lee MS, Kim JY. Sasang types may differ in eating rate, meal size, and regular appetite: a systematic literature review. Asia Pacific Journal of Clinical Nutrition. 2012;21(3):327-337.
41. Rodriguez G, Moreno LA. Is diet the fuel for obesity in children and adolescents? Obesity and Metabolism-Milan. 2008;4(3):183-188.
42. Tsiaousi M, Υanasmidis A. Η Επίδραση των Διατροφικών Συνηθειών και της Φυ. Nosileftiki. 2012;51(2):178-186.
43. Watanabe E, Lee JS, Mori K, Kawakubo K. Clustering patterns of obesity-related multiple lifestyle behaviours and their associations with overweight and family environments: a cross-sectional study in Japanese preschool children. BMJ Open. 2016;6(11):e012773.
44. Waxman M, Stunkard AJ. Caloric intake and expenditure of obese boys. Journal of Pediatrics. 1980;96(2):187-193.
45. Wijtzes AI, Jansen W, Bouthoorn SH, et al. Meal-Skipping Behaviors and Body Fat in 6-Year-Old Children. Journal of Pediatrics. 2016;168:118-125.e112.
46. Wijtzes AI, Jansen W, Jaddoe VWV, et al. Social Inequalities in Young Children's Meal Skipping Behaviors: The Generation R Study. Plos One. 2015;10(7).
47. Yeh S-SS, Brown RF. Disordered eating partly mediates the relationship between poor sleep quality and high body mass index. Eating Behaviors. 2014;15(2):291-297.
48. Yoo S, Kim H, Lee J. Abnormal weight status and associated characteristics of low-income Korean children. Asia-Pacific Journal of Public Health. 2015;27(2):NP1093-1105.
49. Yorulmaz H, Pacal FP. Assessment of Nutritional Habits and Obesity Situations of Adolescents in 16-18 Age Group. Turkiye Klinikleri Tip Bilimleri Dergisi. 2012;32(2):364-370.
50. Bodur S, Uguz M, Sahin N. BEHAVIORAL RISK FACTORS FOR OVERWEIGHT AND OBESITY IN TURKISH ADOLESCENTS. Nobel Medicus. 2010;6(3):79-83.
51. Ciccone J, Woodruff SJ, Fryer K, Campbell T, Cole M. Associations among evening snacking, screen time, weight status, and overall diet quality in young adolescents. Applied Physiology Nutrition and Metabolism-Physiologie Appliquee Nutrition Et Metabolisme. 2013;38(7):789-794.
52. Dubois L, Girard M, Kent MP, Farmer A, Tatone-Tokuda F. Breakfast skipping is associated with differences in meal patterns, macronutrient intakes and overweight among pre-school children. Public Health Nutrition. 2009;12(1):19-28.
53. Gluck ME, Venti CA, Salbe AD, Krakoff J. Nighttime eating: commonly observed and related to weight gain in an inpatient food intake study. American Journal of Clinical Nutrition. 2008;88(4):900-905.
54. Ostachowska-Gasior A, Piwowar M, Kwiatkowski J, Kasperczyk J, Skop-Lewandowska A. Breakfast and Other Meal Consumption in Adolescents from Southern Poland. International Journal of Environmental Research & Public Health [Electronic Resource]. 2016;13(5):28.
55. Prochnik Estima CC, da Costa RS, Sichieri R, Pereira RA, da Veiga GV. Meal consumption patterns and anthropometric measurements in adolescents from a low socioeconomic neighborhood in the metropolitan area of Rio de Janeiro, Brazil. Appetite. 2009;52(3):735-739.
56. Tee JYH, Gan WY, Tan KA, Chin YS. Obesity and unhealthy lifestyle associated with poor executive function among Malaysian adolescents. PLoS ONE [Electronic Resource]. 2018;13(4):e0195934.
57. Xiong LH, Wang CL, Chen ZQ, et al. [Study on food preference and dietary behavior to overweight/obesity in school children and adolescents in Guangzhou: a case-control study]. Chung-Hua Liu Hsing Ping Hsueh Tsa Chih Chinese Journal of Epidemiology. 2008;29(10):965-969.
58. Azadbakht L, Akbari F, Qorbani M, et al. Dinner consumption and cardiovascular disease risk factors among a nationally representative sample of iranian adolescents: The CaSPian-III study. Journal of Cardiovascular and Thoracic Research. 2019;11(2):138-146.
59. Palla L, Almoosawi S. Diurnal Patterns of Energy Intake Derived via Principal Component Analysis and Their Relationship with Adiposity Measures in Adolescents: Results from the National Diet and Nutrition Survey RP (2008-2012). Nutrients. 2019;11(2).
60. Top FU, Kaya B, Tepe B, Cam HH. Prevalence of Obesity and Related Risk Factors among Secondary School Adolescents. International Journal of Caring Sciences. 2019;12(2):994-1000.
61. Vilela S, Oliveira A, Severo M, Lopes C. Chrono-Nutrition: The Relationship between Time-of-Day Energy and Macronutrient Intake and Children's Body Weight Status. Journal of Biological Rhythms. 2019;34(3):332-342.
62. Cezimbra VG, De Oliveira MT, Pereira LJ, et al. Meal intake and overweight in schoolchildren aged 7 to 12 years old in a city in southern brazil. Obesity Facts. 2019;12 (Supplement 1):207.
63. Lioret S, Touvier M, Lafay L, Volatier JL, Maire B. Are eating occasions and their energy content related to child overweight and socioeconomic status? Obesity. 2008;16(11):2518-2523.
64. Musaiger AO, Al-Roomi K, Bader Z. Social, dietary and lifestyle factors associated with obesity among Bahraini adolescents. Appetite. 2014;73:197-204.
65. Onyiriuka AN, Umoru DD, Ibeawuchi AN. Weight status and eating habits of adolescent Nigerian urban secondary school girls. SAJCH South African Journal of Child Health. 2013;7(3):108-112.
66. Munoz, J. S. G., Cañavate, R., Hernandez, C. M., et al. The association among chronotype, timing of food intake and food preferences depends on body mass status. European journal of clinical nutrition. 2017;71(6): 736-742.
67. Ochiai H, Shirasawa T, Ohtsu T, et al. Eating behaviors and overweight among adolescents: a population-based survey in Japan. Journal of Obesity. 2013;2013:717942.
68. Gómez-Martínez S, Martínez-Gómez D, Perez de Heredia F, et al. Eating Habits and Total and Abdominal Fat in Spanish Adolescents: Influence of Physical Activity. The AVENA Study. Journal of Adolescent Health. 2012;50(4):403-409.
69. Vik FN, Bjornara HB, Overby NC, et al. Associations between eating meals, watching TV while eating meals and weight status among children, ages 10-12 years in eight European countries: the ENERGY cross-sectional study. International Journal of Behavioral Nutrition & Physical Activity. 2013;10:58.
70. Reed M, Dancy B, Holm K, Wilbur J, Fogg L. Eating behaviors among early adolescent African American girls and their mothers. Journal of School Nursing. 2013;29(6):452-463.
71. Rychkova LV, Ajurova ZG, Pogodina AV, Kosovtseva AS. Risk factors for obesity in adolescents of ethnic groups in rural areas of the republic of buryatia: a cross-sectional study. [Russian]. Voprosy Sovremennoi Pediatrii - Current Pediatrics. 2017;16(6):509-515.
72. Abd El-Shaheed A, Mahfouz NN, Moustafa RSI, Elabd MA. Alarming eating behaviours among adolescents in Egypt. Open Access Macedonian Journal of Medical Sciences. 2019;7(13):2189-2193.
73. Alavi Naini AM, Amini M, Karajibani M, et al. Association of obesity with food habits and body image in school children of Nakhon Pathom Province, Thailand. Iranian Journal of Public Health. 2006;35(2):42-48.
74. Eloranta AM, Lindi V, Schwab U, et al. Dietary factors associated with overweight and body adiposity in Finnish children aged 6-8 years: the PANIC Study. International Journal of Obesity. 2012;36(7):950-955.
75. Azizi, F., Allahverdian, S., Mirmiran, P., et al. Dietary factors and body mass index in a group of Iranian adolescents: Tehran lipid and glucose study-2. International journal for vitamin and nutrition research, 2001;71(2): 123-127.
76. Huang Y, Ho SY, Huang R, Lo WS, Lam TH. Night eating in Hong Kong adolescents: prevalence and associations with dinner habits, bedtime and weight status. The University of Hong Kong (Pokfulam, Hong Kong), 2014.
77. Sun M, Hu X, Li F, Deng J, Shi J, Lin Q. Eating Habits and Their Association with Weight Status in Chinese School-Age Children: A Cross-Sectional Study. Int J Environ Res Public Health, 2020;17(10): pp.3571.
78. Agustina R, Nadiya K, Andini EA., et al. Associations of meal patterning, dietary quality and diversity with anemia and overweight-obesity among Indonesian school-going adolescent girls in West Java. PloS one, 2020;15(4): e0231519.
79. Abduelkarem, A. R., Sharif, S. I., Bankessli, F. G., et al. Obesity and its associated risk factors among school-aged children in Sharjah, UAE. PloS one, 2020;15(6): e0234244.
