# Supplementary material for: The association between later eating rhythm and adiposity in children and adolescents: a systematic review and meta-analysis
Source: Nutr Rev. 2022 May 4;80(6):1459–79. doi: 10.1093/nutrit/nuab079 (PMC9086801; doi:10.1093/nutrit/nuab079)
Supplement: nuab079_Supplementary_Data [file nuab079_supplementary_data.zip › Zou_Sensitivity analysis on dietary measurements_file S3.pdf]

File S3 Sensitivity analysis excluding studies using less reliable dietary measurements

Table Differences in the association between exposure and adiposity between studies using more reliable dietary assessment and corresponding review results.

Study	Exposure	Dietary assessment	Quality score	Study result	Review result	Difference
*Coulthard et al. (2016)[S1]	**Timing:** Evening meal after 8pm	4-day food diary	Medium (7/10)	Children: no association Adolescents: no association	No association	No
Eloranta et al. (2012)[S2]	**Energy intake for:** evening main meal	4-day food diary	High (8/10)	Children: no association	No association	No
Thompson et al. (2006)[S3]	**Energy intake for:** whole evening period	7-day food diary	Medium (4/9)	Children: positive association	Children: positive association Adolescents: no association	No
*Vilela et al. (2019)[S4]	**Energy intake for:** around bedtime	3-day food diary	High (7/9)	Children: positive association	Positive association	No
Lioret et al. (2008)[S5]	**Meal frequency:** evening meal skipping	7-day food diary	Medium (5/10)	Children: no association	Children: no association Adolescents: positive association	No

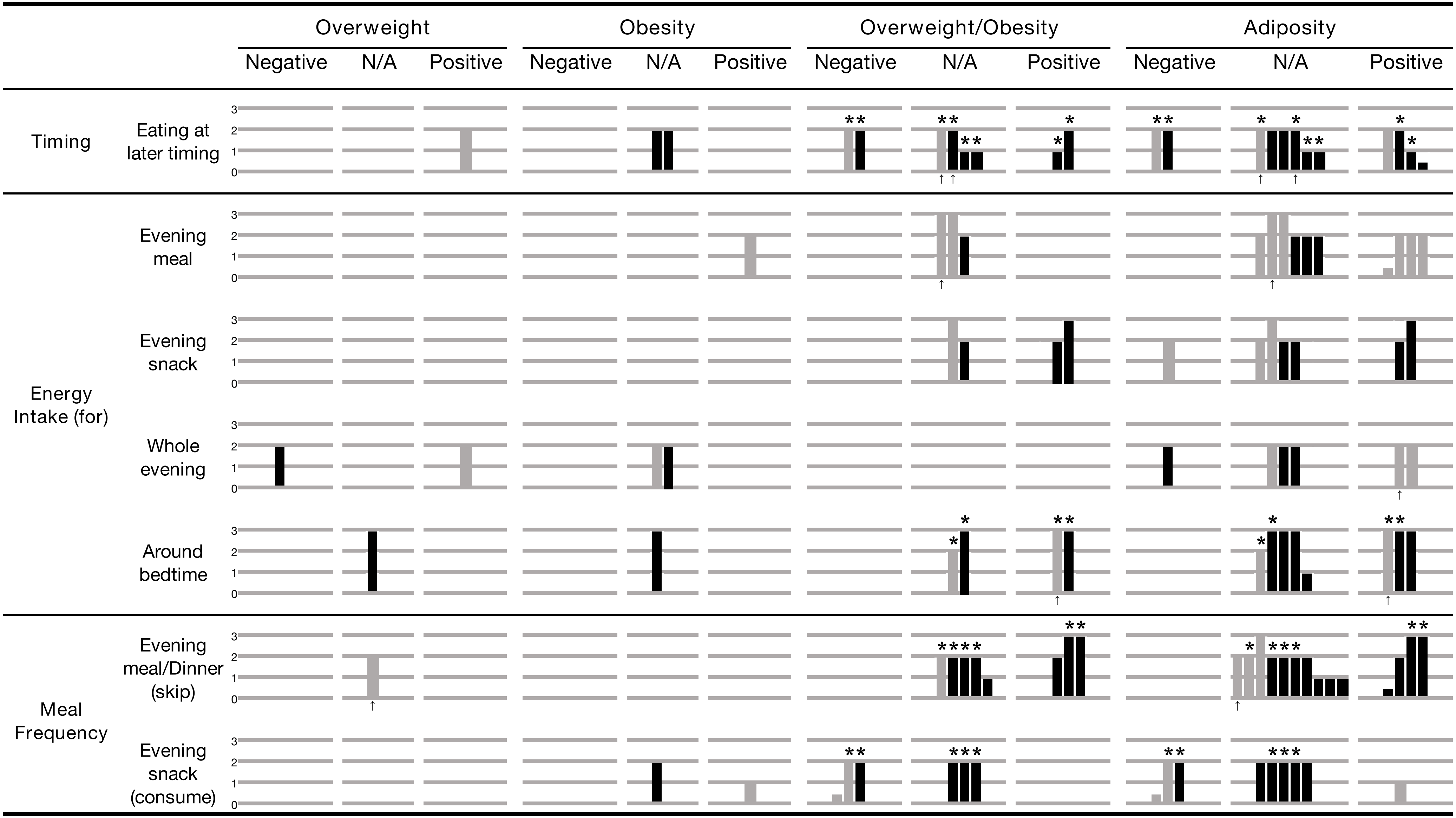

A 'supermatrix' covering all categories of exposures consisting of seven rows (each represents a dimension of later eating rhythm which is commonly described from three aspects, timing, energy intake and meal frequency) and three columns (each represents the direction of the association between one of the indicators of later eating rhythm and weight status, one for a negative association, one for no association (N/A) and one for a positive association). Each bar represents an association between later eating rhythm and weight status. Studies conducted among children (<=10 yrs) population are indicated with half-tone (grey) bars and studies conducted among adolescents (>10 yrs) are indicated with full-tone (black) bars. The quality of each study is indicated by the height of the bar (3=high quality; 2=medium quality; 1=low quality; and 0.5=abstract/conference paper). The studies included in meta-analysis are annotated with a star on the top of each bar. The studies using food record/recall for at least 3 days as dietary measurement are annotated with an arrow at the bottom of each bar.

Supplementary References

S1. Coulthard JD, Pot GK. The timing of the evening meal: how is this associated with weight status in UK children?. Brit J Nutr, 2016;115(9), pp.1616-1622.

S2. Eloranta AM, Lindi V, Schwab U, et al. Dietary factors associated with overweight and body adiposity in Finnish children aged 6–8 years: the PANIC Study. Int J Obes (Lond), 2012;36(7): pp.950-955.

S3. Thompson OM, Ballew C, Resnicow K, et al. Dietary pattern as a predictor of change in BMI z-score among girls. Int J Obes (Lond), 2006;30(1): pp.176-182.

S4. Vilela S, Oliveira A, Severo M, Lopes C. Chrono-Nutrition: The Relationship between Time-of-Day Energy and Macronutrient Intake and Children's Body Weight Status. J Biol Rhythms, 2019;34(3): pp.332-342.

S5. Lioret S, Touvier M, Lafay L, Volatier JL, Maire B. Are eating occasions and their energy content related to child overweight and socioeconomic status?. Obes, 2008;16(11): pp.2518-2523.
