# Supplementary material for: The association between later eating rhythm and adiposity in children and adolescents: a systematic review and meta-analysis
Source: Nutr Rev. 2022 May 4;80(6):1459–79. doi: 10.1093/nutrit/nuab079 (PMC9086801; doi:10.1093/nutrit/nuab079)
Supplement: nuab079_Supplementary_Data [file nuab079_supplementary_data.zip › Zou_Sensitivity analysis on the relationship between eating at later timing_figure S2.docx]

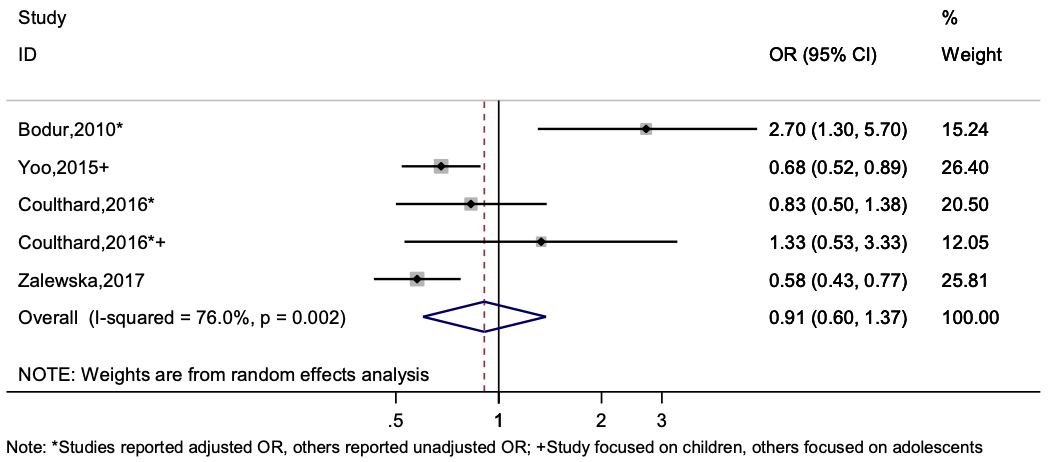


**Figure S2 Sensitivity analysis on the relationship between eating at later timing (after 8pm in general) and adiposity after dropping low quality studies (scored less than 4).**
