# Supplementary material for: The association between later eating rhythm and adiposity in children and adolescents: a systematic review and meta-analysis
Source: Nutr Rev. 2022 May 4;80(6):1459–79. doi: 10.1093/nutrit/nuab079 (PMC9086801; doi:10.1093/nutrit/nuab079)
Supplement: nuab079_Supplementary_Data [file nuab079_supplementary_data.zip › Zou_Summary of results by adjustment for confounders_table S5.pdf]

Table S5. Summary of results (associations between later eating rhythm and adiposity) by adjustment for confounders within each exposure groups.

Study	Study design	Age	No adjustment	Confounders in adjustment											Unadjusted results	Adjusted results
				Age	Sex	Race	SEC	PA	TEI	MR	SQ	Base-line BMI	PEB	other		
Timing-any intake at later timing																
Watanabe et al. (2016)[S1]	CS	Children													n/a	Positive
*Coulthard et al. (2016)[S2]	CS	Children; Adol													n/a	No association; No association
*Bodur et al. (2010)[S3]	CS	Adol								¶				Cookie; Fruit	Positive	Positive
*Yoo et al. (2015)[S4]	CS	Children													Negative	Negative
Alavi Naini et al. (2006)[S5]	CC	Adol													No association	
*Choi et al. (2017)[S6]	CS	Adol													No association	
*El-Shaheed et al. (2019)[S7]	CC	Adol													No association	
Rychkova et al. (2019)[S8]	CC	Adol													No association	
*Dmitruk et al. (2018)[S9]	CS	Adol													Positive	
Huang et al. (2014)[S10]	CS-Abs	Adol													Positive	
*Zalewska& Maciorkowska (2017)[S11]	CS	Adol													Negative	
Barbu et al. (2015)[S12]	CS	Children/ Adol													n/a	
Ayine et al. (2018) [S13]	Abs	Children													n/a	
Total															No association 4/9<br>Positive 3/9	No association 2/5<br>Positive 2/5

Study	Study design	Age	No adjustment	Confounders in adjustment											Unadjusted results	Adjusted results
				Age	Sex	Race	SEC	PA	TEI	MR	SQ	Base line BMI	PEB	other		
Energy intake around bedtime																
*Hernandez et al. (2016)[S14]	CS	Adol												Stress level	OW/OB: No association	OW: No association OB: No association BMI z-score: Positive
*Bo et al. (2014)[S15]	CS	Adol													No association	Positive
*Vilela et al. (2019)[S16]	Cohort	Children												Maternal age;	Positive	Positive
*Lamerz et al. (2005)[S17]	CS	Children													No association	
Yüksel et al. (2017)[S18]	CS	Adol													No association	
Energy intake for evening main meal																
Karatzi et al. (2017)[S19]	CS	Adol												Tanner stage	n/a	No association
Eloranta et al. (2012)[S20]	CS	Children													No association	No association
*Vilela et al. (2019)[S16]	Cohort	Children													No association	
Fayet et al. (2012)[S21]	CS	Children/ Adol													M: No association F : No association	
Azizi F et al. (2001)[S22]	CS	Adol (M;F)													No association	
Maffeis et al. (2000)[S23]	CS	Children													Positive	
Mayorga et al. (2017)[S24]	CS-Abs	Children													Positive	
Waxman & Stunkard (1980)[S25]	CC	Children/ Adol													Positive	
Dubois et al. (2009)[S26]	CS	Children													Positive	

Energy intake for evening snack																
*Bo et al. (2014)[S15]	CS	Adol													No association	Positive
*Vilela et al. (2019)[S16]	Cohort	Children													No association	
Fayet et al. (2012)[S21]	CS	Children/<br>adol													No association	
Maffeis et al. (2000)[S23]	CS	Children<br>(M;F)													M: Negative<br>F: No association	
Azizi F et al. (2001)[S22]	CS	Adol<br>(M;F)													M: No association<br>F: Positive	
Energy intake for whole evening																
Thompson et al. (2006)[S27]	Cohort	Children													n/a	Positive
Eng et al. (2009)[S28]	CS	Children;<br>Adol													n/a	OW:<br>Children: Positive<br>Adol: Negative<br>OB:<br>Children:<br>No association<br>Adol:<br>No association
Karatzi et al. (2017)[S19]	CS	Adol												Tanner stage	n/a	No association
Total															No association<br>14/21<br>Positive<br>6/21	No association<br>7/13<br>Positive<br>6/13

Study	Study design	Age	No adjustment	Confounders in adjustment: Age	Sex	Race	SEC	PA	TEI	MR	SQ	Base line BMI	PEB	other	Unadjusted results	Adjusted results
**Evening meal skipping**																
Taib et al. (2014)[S29]	CS-Abs	Adol				■	■								n/a	Positive
*De Cnop et al. (2018)[S30]	CS	Adol		■	■										n/a	Public school: No association Private school: No association
Gomez-Martin et al. (2012)[S31]	CS	Adol		■				■							n/a	No association
Lehto et al. (2011)[S32]	CS	Children		■	■		■	■			■	■			n/a	No association
Ostachowska-Gasior (2016)[S33]	CS	Adol		■	■					■ ¶ §				Dessert	n/a	No association
*Wijtzes et al. (2016)[S34]	Cohort	Children		■	■	■	■			■		■		Parental BMI	OW/OB: Positive	OW/OB: No association Fat mass: No association
*Agustina et al. (2020)[S35]	CS	Adol					■		■					Radio	Wekday: positive Weekend: No association	Weekday: positive Weekend: No association
*Azadbakht et al. (2019)[S36]	CS	Adol		■	■		■	■						Smoking	BMI: Positive OW/OB: Positive Abdominal OB: Positive	OW/OB: Positive Abdominal OB: Positive
Vik et al. (2013)[S37]	CS	Adol	■												Positive	
*Zalewska& Maciorkowska (2017)[S11]	CS	Adol	■												No association	
Yorulmaz et al. (2012)[S38]	CS	Adol	■												No association	
Lioret et al. (2008)[S39]	CS	Children	■												No association	
*Musaiger et al. (2014)[S40]	CS	Adol	■												No association	
Reed et al. (2013)[S41]	CS	Adol	■												No association	

Total															No association 6/12<br>Positive 6/12	No association 8/12<br>Positive 4/12
Evening snack consumption																
Gomez-Martin et al. (2012)[S31]	CS	Adol													n/a	No association
Ben Slama et al. (2002)[S42]	CC	Children													Positive	
Cezimbra et al. (2019)[S43]	CS-Abs	Children													Negative	
*Top et al. (2019)[S44]	CS	Adol													Negative	
*Ciccone et al. (2013)[S45]	CS	Children													Negative	
Rychkova et al. (2019)[S8]	CC	Adol													No association	
*Musaiger et al. (2014)[S40]	CS	Adol													No association	
*Ochiai et al. (2013)[S46]	CS	Adol													No association	
*Sun et al. (2020)[S47]	CS	Adol													No association	
Total															No association 4/8<br>Negative 3/8	No association 1/1

*studies included in meta-analyses; CC, case control study; CS, cross-sectional study; Abs, abstract; Adol, adolescents; M, male; F, female; BMI, body mass index; SEC, socioeconomic status; PA, physical activity; TEI, total energy intake; MR, meal regularity; ¶ breakfast skipping; § morning snack skipping; SQ, sleep quality; PEB, parental eating behaviour; OW, overweight; OB, obesity; n/a, not available.

Supplementary References

S1. Watanabe E, Lee JS, Mori K, Kawakubo K. Clustering patterns of obesity-related multiple lifestyle behaviours and their associations with overweight and family environments: a cross-sectional study in Japanese preschool children. BMJ open, 2016;6(11).

S2. Coulthard JD, Pot GK. The timing of the evening meal: how is this associated with weight status in UK children?. Brit J Nutr, 2016;115(9), pp.1616-1622.

S3. Bodur S, Uguz M, Şahin N. Behavioral risk factors for overweight and obesity in Turkish adolescents. Emerg, 2010;20: p.23.

S4. Yoo S, Kim H, Lee J. Abnormal Weight Status and Associated Characteristics of Low-Income Korean Children. Asia-Pac J Public He, 2015;27(2): pp.NP1093-NP1105.

S5. Alavi NA, Amini M, Karajibani M, et al. Association of obesity with food habits and body image in school children of Nakhon Pathom Province, Thailand, 2006.

S6. Choi MK, Cho YJ, Kim MH, Bae YJ. Night eating status according to body mass index of Korean adolescents. Nutr Food Sci. 2017.

S7. Abd El-Shaheed A, Mahfouz NN, Moustafa RS, Elabd MA. Alarming Eating Behaviours among Adolescents in Egypt. Open Access Maced J Med Sci, 2019;7(13): p.2189.

S8. Rychkova L, Pogodina A, Ayurova Z, Berdina O. Risk Factors for Obesity in Adolescents Living in Rural Areas of Buryatia: A Case-Control Study. J Biomed, 2019;9(2): pp.190-195.

S9. Dmitruk A, Kunicka I, Poplawska H, Holub W. Dietary patterns among girls aged 16-18 years old according to their body mass index and waist-to-hip ratio. Pediatria I Med Rodz,2018;14(1): pp.78-87.

S10. Huang Y, Ho SY, Huang R, Lo WS, Lam TH. Night eating in Hong Kong adolescents: prevalence and associations with dinner habits, bedtime and weight status. The University of Hong Kong (Pokfulam, Hong Kong), 2014.

S11. Zalewska M, Maciorkowska E. Selected nutritional habits of teenagers associated with overweight and obesity. PeerJ, 2017;5, p.e3681.

S12. Barbu CG, Teleman MD, Albu AI, et al. Obesity and eating behaviors in school children and adolescents–data from a cross sectional study from Bucharest, Romania. BMC Public Health, 2015;15(1): p.206.

S13. Ayine P, Parra EP, Jeganathan RB, Thangiah G. Influence of Race, Ethnicity, and Behavioral Factors on Childhood Obesity. 2018.

S14. Hernandez E, Kim M, Kim WG, Yoon J. Nutritional aspects of night eating and its association with weight status among Korean adolescents. Nutr Res Pract, 2016;10(4): pp.448-455.

S15. Bo S, De Carli L, Venco E, et al. Impact of snacking pattern on overweight and obesity risk in a cohort of 11-to 13-year-old adolescents. J Pediatr Gastroenterol Nutr, 2014;59(4), pp.465-471.

S16. Vilela S, Oliveira A, Severo M, Lopes C. Chrono-Nutrition: The Relationship between Time-of-Day Energy and Macronutrient Intake and Children's Body Weight Status. J Biol Rhythms, 2019;34(3): pp.332-342.

S17. Lamerz A, Kuepper-Nybelen J, Bruning N, et al. Prevalence of obesity, binge eating, and night eating in a cross-sectional field survey of 6-year-old children and their

parents in a German urban population. J Child Psychol Psychiatry, 2005;46(4):pp.385-393.

S18. Yüksel A, Önal HY, Kurt KG. Adherence to the Mediterranean diet and factors affecting obesity in high school students. Int J Med Sci Public Health, 2017;6(12): pp.78-86.

S19. Karatzi K, Moschonis G, Choupi E, et al. Late-night overeating is associated with smaller breakfast, breakfast skipping, and obesity in children: The Healthy Growth Study. Nutrition, 2017;33: pp.141-144.

S20. Eloranta AM, Lindi V, Schwab U, et al. Dietary factors associated with overweight and body adiposity in Finnish children aged 6–8 years: the PANIC Study. Int J Obes (Lond), 2012;36(7): pp.950-955.

S21. Fayet F, Mortensen A, Baghurst K. Energy distribution patterns in Australia and its relationship to age, gender and body mass index among children and adults. Nutr Diet, 2012;69(2): pp.102-110.

S22. Azizi F, Allahverdian S, Mirmiran P, Rahmani M, Mohammadi F. Dietary factors and body mass index in a group of Iranian adolescents: Tehran lipid and glucose study-2. Int J Vitam Nutr Res, 2001;71(2): pp.123-127.

S23. Maffeis C, Provera S, Filippi L, et al. Distribution of food intake as a risk factor for childhood obesity. Int J Obes (Lond), 2000;24(1): pp.75-80.

S24. Mayorga Mazon CDLM, Monzon Rodriguez AN, Ligerini Vazquez LJ, Menendez Blanco CY, Guerendiain Margni ME. Energy, Protein and carbohydrate intake in relation to anthropometric parameters at different meal times in children of evanes study. Ann Nutr Metab, 2017;71: pp. 609-609.

S25. Waxman M, Stunkard AJ. Caloric intake and expenditure of obese boys. J Pediatr, 1980;96(2): pp.187-193.

S26. Dubois L, Girard M, Kent MP, Farmer A, Tatone-Tokuda F. Breakfast skipping is associated with differences in meal patterns, macronutrient intakes and overweight among pre-school children. Public Health Nutr, 2009;12(1): pp.19-28.

S27. Thompson OM, Ballew C, Resnicow K, et al. Dietary pattern as a predictor of change in BMI z-score among girls. Int J Obes (Lond), 2006;30(1): pp.176-182.

S28. Eng S, Wagstaff DA, Kranz S. Eating late in the evening is associated with childhood obesity in some age groups but not in all children: the relationship between time of consumption and body weight status in US children. Int J Behav Nutr Phy, 2009;6(1): p.27.

S29. Taib M, Chin Y, Wahida F, Kaartina S, Woon F, Zalilah M. Meal skipping as a risk factor of abdominal obesity among Malaysian adolescents: findings from the Malaysian overweight and disordered eating survey: T3: S14. 35. Obes Rev, 2014;15.

S30. De Cnop ML, Monteiro LS, Rodrigues PRM, Estima CCP, da Veiga GV, Pereira RA. Meal habits and anthropometric indicators in adolescents from public and private schools of the metropolitan region of Rio de Janeiro/Consumo de refeicoes e indicadores antropometricos em adolescentes de escolas publicas e privadas da regiao metropolitana do Rio de Janeiro. Rev de Nutr, 2018;31(1): pp.35-48.

S31. Gómez-Martínez S, Martínez-Gómez D, de Heredia FP, et al. Eating habits and total and abdominal fat in Spanish adolescents: influence of physical activity. The AVENA study. J Adolesc Health, 2012;50(4): pp.403-409.82.

S32. Lehto R, Ray C, Lahti-Koski M, Roos E. Meal pattern and BMI in 9–11-year-old children in Finland. Public Health Nutr, 2011;14(7), pp.1245-1250.
S33. Ostachowska-Gasior A, Piwowar M, Kwiatkowski J, Kasperczyk J, Skop-Lewandowska A. Breakfast and other meal consumption in adolescents from southern Poland. Int J Environ Res Public Health, 2016;13(5): p.453.
S34. Wijtzes AI, Jansen W, Bouthoorn SH, et al. Meal-skipping behaviors and body fat in 6-year-old children. Journal pediatr, 2016;168: pp.118-125.
S35. Agustina R, Nadiya K, Andini EA., et al. Associations of meal patterning, dietary quality and diversity with anemia and overweight-obesity among Indonesian school-going adolescent girls in West Java. PloS one, 2020;15(4): e0231519.
S36. Azadbakht L, Akbari F, Qorbani M, et al. Dinner consumption and cardiovascular disease risk factors among a nationally representative sample of Iranian adolescents: the CASPIAN-III Study. J Cardiovasc Thorac Res, 2019;11(2): p.138.
S37. Vik FN, Bjørnarå HB, Øverby NC, et al. Associations between eating meals, watching TV while eating meals and weight status among children, ages 10–12 years in eight European countries: the ENERGY cross-sectional study. Int J Behav Nutr Phys Act, 2013;10(1): p.58.
S38. Yorulmaz H, Pacal FP. Assessment of nutritional habits and obesity situations of adolescents in 16-18 age group. Turk Klin Rip Bilim, 2012;32(2): pp.364-370.
S39. Lioret S, Touvier M, Lafay L, Volatier JL, Maire B. Are eating occasions and their energy content related to child overweight and socioeconomic status?. Obes, 2008;16(11): pp.2518-2523.
S40. Musaiger AO, Al-Roomi K, Bader Z. Social, dietary and lifestyle factors associated with obesity among Bahraini adolescents. Appetite, 2014;73: pp.197-204.
S41. Reed M, Dancy B, Holm K, Wilbur J, Fogg L. Eating behaviors among early adolescent African American girls and their mothers. J Sch Nurs, 2013;29(6): pp.452-463.
S42. Ben Slama F, Achour A, Belhadj O, Hsairi M, Oueslati M, Achour N. Obesity and way of life in a schoolboy population of the Ariana region (Tunisia) aged of 6 to 10 years. Tunis Med, 2002;80(9): pp.542-547.
S43. Cezimbra VG, De Oliveira MT, Pereira LJ, et al. Meal intake and overweight in schoolchildren aged 7 to 12 years old in a city in southern brazil. Obes Facts, 2019;12 (Supplement 1): p. 207.
S44. Kaya Band, Tepe B. Prevalence of Obesity and Related Risk Factors among Secondary School Adolescents. Int J Caring Sci, 2019;12(2): pp.1-7.
S45. Ciccone J, Woodruff SJ, Fryer K, Campbell T, Cole M. Associations among evening snacking, screen time, weight status, and overall diet quality in young adolescents. Appl Physiol Nutr Metab, 2013;38(7): pp.789-794.
S46. Ochiai H, Shirasawa T, Ohtsu T, et al. Eating behaviors and overweight among adolescents: a population-based survey in Japan. J Obes, 2013.
S47. Sun M, Hu X, Li F, Deng J, Shi J, Lin Q. Eating Habits and Their Association with Weight Status in Chinese School-Age Children: A Cross-Sectional Study. Int J Environ Res Public Health, 2020;17(10): pp.3571.
