# Supplementary material for: The association between later eating rhythm and adiposity in children and adolescents: a systematic review and meta-analysis
Source: Nutr Rev. 2022 May 4;80(6):1459–79. doi: 10.1093/nutrit/nuab079 (PMC9086801; doi:10.1093/nutrit/nuab079)
Supplement: nuab079_Supplementary_Data [file nuab079_supplementary_data.zip › Zou_The certainty of evidence according to GRADE_table S4.pdf]

Table S4. The certainty of evidence of the associations between later eating rhythm (four distinct aspects) and overweight/obesity according to GRADE.

Exposure	Outcome	Studies n	Participants n	Anticipated absolute effects* (95%CI)		OR (95%CI)	Certainty of the evidence (GRADE)	Comments
				Risk of adiposity in control group	Risk of adiposity in exposure group			
Eating at later timing	Overweight/obesity	7[a]	5263	205 per 1,000	211 per 1,000 (149 to 293)	1.04 (0.68 to 1.61)	Very Low due to observation design, RoB, inconsistency, indirectness, imprecision.※	The true effect is likely to be substantially different from the estimate of effect
Higher energy intake around bedtime	Overweight/obesity	4	4130	129 per 1,000	149 per 1,000 (135 to 164)	1.19 (1.06 to 1.33)	Very Low due to observation design, inconsistency, indirectness, imprecision.§	The true effect is likely to be substantially different from the estimate of effect
Evening main meal skipping	Overweight/obesity	6[b]	8487	282 per 1,000	338 per 1,000 (309 to 368)	1.30 (1.14 to 1.48)	Very Low due to observation design, imprecision.⁋	The true effect is likely to be substantially different from the estimate of effect
Evening snack consumption	Overweight/obesity	5	7747	265 per 1,000	224 per 1,000 (182 to 274)	0.80 (0.62 to 1.05)	Very Low due to observation design, RoB, inconsistency, imprecision.†	The true effect is likely to be substantially different from the estimate of effect

GRADE: Grading of Recommendations Assessment, Development and Evaluation; OR: Odds ratio; CI: Confidence interval; RoB: Risk of bias.

*The risk in the intervention group (and its 95% confidence interval) is based on the assumed risk in the comparison group and the relative effect of the exposure (and its 95% CI).

a. 8 datasets from 7 studies; b. 8 datasets from 6 studies.

※Evidence start with Low, but downgraded to Very Low due to RoB (three of seven studies scored low quality in the quality assessment), inconsistency (variation in CI, I2=82.6%,P<0.001 for heterogeneity), indirectness (adjusted and unadjusted combined; different definition of "later timing"; different populations; case control and cross sectional combined), and imprecision (includes null effect AND appreciable benefit or harm).

§Evidence start with Low, but downgraded to Very Low due to inconsistency (variation in CI), indirectness (adjusted result and unadjusted result combined; cohort study and cross sectional study combined), imprecision (includes null effect AND appreciable benefit or harm).

⁋Evidence start with Low, but downgraded to Very Low due to imprecision (includes null effect AND appreciable benefit or harm).

†Evidence start with Low, but downgraded to Very Low due to RoB (all studies scored medium quality in the quality assessment, no high-quality study), inconsistency (no overlap of CIs, I2 =73%, P=0.005 for heterogeneity), and imprecision (includes null effect AND appreciable benefit or harm).
