# Supplementary figures and images for: The association between later eating rhythm and adiposity in children and adolescents: a systematic review and meta-analysis
Source: Nutr Rev. 2022 May 4;80(6):1459–79. doi: 10.1093/nutrit/nuab079 (PMC9086801; doi:10.1093/nutrit/nuab079)

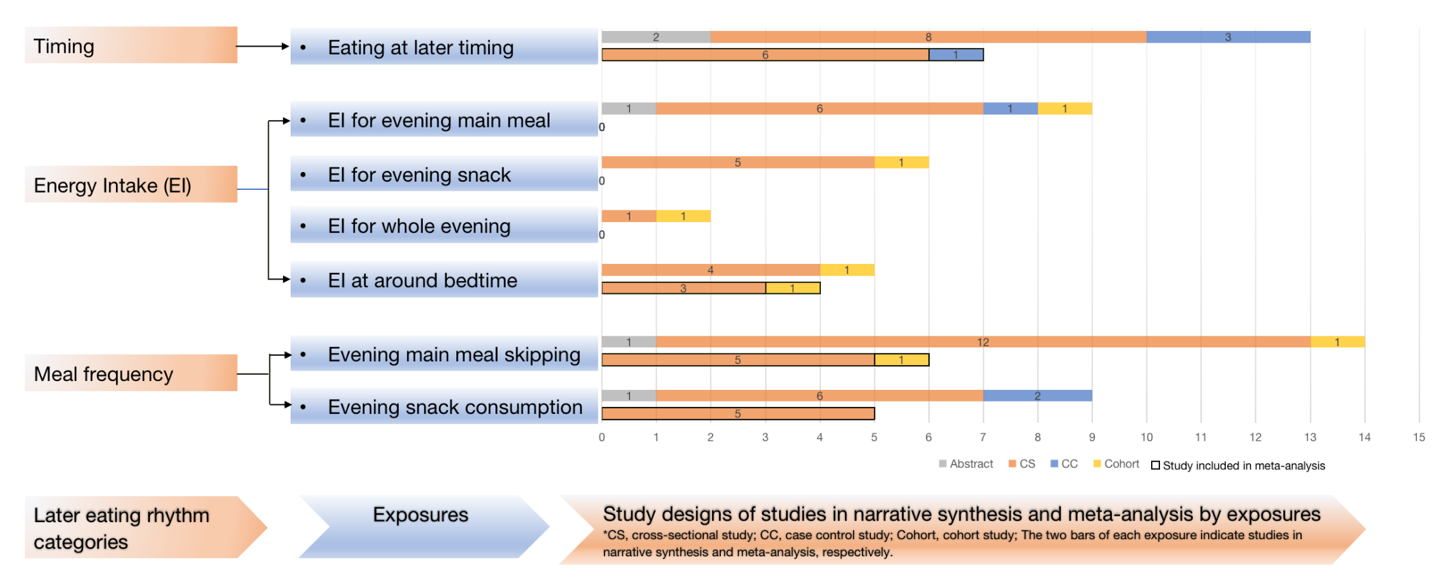


**Figure S1 Study design of studies included in each exposure group.**

Supplement: nuab079_Supplementary_Data [file nuab079_supplementary_data.zip › Zou_Study design of studies included in each exposure group_figure S1.docx]
